# Time-varying associations between corticosteroid dose and hospital mortality in ARDS: a sliding-window analysis of MIMIC-IV

**DOI:** 10.1186/s12890-026-04251-w

**Published:** 2026-03-26

**Authors:** Dominic C. Marshall, Matthieu Komorowski, Matthieu Jamme, Brijesh V. Patel, David B. Antcliffe, Sonali Parbhoo

**Affiliations:** 1 Department of Surgery and Cancer, Division of Anaesthetics, Pain Medicine and Intensive Care, Imperial College London, London, UK; 2 Cleveland Clinic London, London, UK; 3 Charing Cross Hospital, London, UK; 4 Intensive Care Unit, West Parisian Private Hospital, Ramsay-Générale de Santé, Trappes, France; 5 School of Electrical and Electronic Engineering, Imperial College London, London, UK

**Keywords:** ARDS, Corticosteroids, Dose, Timing, Sliding window, Augmented inverse probability weighting, Overlap weights, MIMIC-IV, Observational study

## Abstract

**Background:**

Corticosteroids are now recommended in guidelines for patients with acute respiratory distress syndrome (ARDS); however, optimal timing and dose remain uncertain. We assessed whether the association between corticosteroids and hospital mortality varies over time in the ICU.

**Methods:**

We performed a retrospective observational study of ARDS patients identified in the MIMIC-IV database (2008–2019). To analyze the time-varying association between corticosteroids and hospital mortality, we constructed overlapping three-day windows from ARDS days 0 to 14. We compared windows with no corticosteroid exposure (0 mg) to windows meeting cumulative prednisolone-equivalent dose thresholds chosen to approximate regimens from landmark clinical trials (≥ 30, ≥ 150, ≥270, ≥ 390 mg PE over 3 days). We estimated overlap-weighted, doubly robust adjusted risk differences (OWRD) for hospital mortality using augmented inverse probability weighting (AIPW).

**Results:**

Of 987 included patients, 354 (35.9%) received corticosteroids, with 262 (74.1%) and 128 (36.2%) of treated patients meeting the ≥ 150 mg and ≥ 390 mg thresholds in at least one window. Early, low cumulative dosing (≥ 30 mg/3d) was not associated with a detectable difference in hospital mortality (e.g., days 0–2: OWRD 0.03, 95% CI − 0.05 to 0.10). Conversely, higher cumulative doses received later in the ICU stay (≥ 150–390 mg/3d after day 8) were associated with higher observed mortality (e.g., days 8–10: OWRD 0.25, 95% CI 0.11–0.39). However, estimates in late, high-dose windows were less precise due to limited covariate overlap and smaller sample sizes.

**Conclusions:**

In unselected ARDS, we found no evidence of benefit or harm from early lower-dose corticosteroids, but higher cumulative doses later in ICU stay were associated with higher mortality, possibly reflecting residual confounding and limited covariate overlap. These hypothesis generating findings support randomized studies testing corticosteroid timing and dose in ARDS.

**Supplementary Information:**

The online version contains supplementary material available at 10.1186/s12890-026-04251-w.

## Introduction

Acute respiratory distress syndrome (ARDS) is a heterogeneous clinical syndrome characterized by acute hypoxaemic respiratory failure and non-cardiogenic pulmonary oedema, accounting for approximately 10% of ICU admissions and substantial mortality [1]. The pathophysiology of ARDS involves a complex interplay of inflammatory and fibroproliferative processes, with dysregulated inflammation driving alveolar-capillary barrier disruption and impaired gas exchange [2]. Importantly, inflammation evolves over time and likely differs across patients, raising critical questions about the timing of immunomodulatory therapy.

Corticosteroids act via glucocorticoid receptor–mediated suppression of inflammatory signalling and transcriptional programs [3, 4] and are now recommended in clinical ARDS guidelines [5]. The optimal timing of corticosteroid administration in ARDS remains a subject of debate [6]. Early intervention, aimed at attenuating the initial inflammatory surge, has been associated with improved outcomes [7–9]. Conversely, initiating corticosteroids later in the disease course may be associated with increased mortality due to irreversible tissue damage and neuromyopathy [7]. 

Observational evaluations of corticosteroid timing and dose are vulnerable to time-varying confounding by indication: clinicians initiate or escalate corticosteroids in response to evolving severity and treatment response. To investigate this, we used an overlapping sliding-window approach paired with doubly robust confounding adjustment (augmented inverse probability weighting (AIPW)) to describe how the adjusted association between corticosteroid exposure and hospital mortality varies with time from ARDS onset [8–10]. 

Our primary objective was to estimate window-specific adjusted risk differences in hospital mortality comparing corticosteroid exposure versus no exposure across days 0–14 after ARDS onset, and across clinically motivated cumulative dose thresholds. To do this, we analysed a sequence of overlapping 3-day windows from ARDS onset to describe how the adjusted association between corticosteroid exposure and hospital mortality varies over time.

## Methods

### Data source

We extracted data from the MIMIC-IV (Version 1.0) database populated by patients admitted to one of the ICUs in the Beth Israel Deaconess Medical Center between 2008 and 2019. Data were extracted via SQL from the Google Cloud Platform BigQuery [11]. 

### Subject selection

We identified mechanically ventilated patients with PaO₂/FiO₂ (P/F) < 300 mmHg and PEEP ≥ 5 cmH₂O for 3 days (or 2 days if death occurred on day 3). This three-day persistence requirement was used to increase diagnostic specificity and ensure adequate clinical data for ARDS adjudication (Methods S1). Within this group ARDS was confirmed using the Berlin definition by chart review (discharge summaries, radiology, echocardiography) [12]. Full cohort flow and criteria are in Methods S1 (Fig. S1).

### Data pre-processing

We constructed overlapping three-day windows (days 0–2, 1–3, …, 12–14) relative to ARDS onset (time zero). For each window we computed summary statistics (e.g., mean, range, variance) for dynamic variables across the three days. Corticosteroid administrations (oral or intravenous) were recorded in 24-hour bins and converted to prednisolone-equivalent (PE) doses using standard equivalence Table [13]. We used last-observation-carried-forward up to 48 h for laboratory variables other than arterial blood gas results. Remaining missing data were imputed using multiple imputation using chained equations (details in Methods S2).

### Sliding time window and corticosteroid dose thresholds

We implemented an overlapping sliding-window (landmark-type) design with 3‑day windows (days 0–2, 1–3, …, 12–14) after ARDS onset. In each window, eligible patients contributed one observation for that window. Exposure was defined by cumulative prednisolone-equivalent corticosteroid dose during the 3‑day window meeting a pre-specified threshold. The comparator group was windows with 0 mg corticosteroid exposure. Because exposure is defined over a 3‑day assessment period, window-specific analyses are conditional on patients contributing data for that window (i.e., remaining under observation during that period), and we interpret results as adjusted associations rather than causal effects.

Corticosteroid dose thresholds were tested based on total corticosteroid received over a three-day time window:

- “Any corticosteroid”: ≥ 30 mg PE over the 3-day window (i.e., > 10 mg/day), excluding physiologic replacement doses.
- ≥ 150 mg/3d PE (~200 mg/day hydrocortisone equivalent to doses used in CAPE-COD) [14].
- ≥ 270 mg/3d PE (~70 mg/day methylprednisolone ≈ 1 mg/kg/day for a 70 kg patient).
- ≥ 390 mg/3d PE (~20 mg/day dexamethasone equivalent to doses used in DEXA-ARDS) [15].

To ensure a clear contrast at each threshold, we excluded windows in which patients received some corticosteroid but did not reach the threshold (0 < dose< threshold). Thus, each comparison contrasts 0 mg versus ≥threshold mg within the same 3-day window.

### Outcome

The prespecified primary outcome was all-cause hospital mortality.

### Covariates and adjustment set

A prespecified directed acyclic graph (DAG) (Fig. S2) and clinical expertise defined ~ 280 candidate covariates. To reduce redundancy, we iteratively pruned covariates using domain expertise, model importance, and diagnostics until model performance deteriorated. The propensity score (PS) model used ~ 25 covariates per window (including corticosteroid history terms) drawn from baseline characteristics and window-level summaries of dynamic physiologic/laboratory variables; the outcome model used a partially overlapping set (Methods S3).

### Model specification and AIPW estimation procedure

The primary estimand was the overlap-weighted, AIPW-adjusted risk difference (OWRD) in hospital mortality. Overlap weighting down-weights extreme propensity scores and emphasizes patients with better covariate overlap between exposure groups, improving stability when positivity is limited. Put simply, OWRD focuses inference on patients with good overlap between exposure groups, estimating the adjusted absolute difference in observed hospital mortality risk between exposure states within each window.

We estimated OWRD using a doubly robust augmented inverse-probability-weighted estimator (Methods S4). For each patient, we combined (i) an IPW term with overlap weights $\:w\left(x\right)\propto\:e\left(x\right)\left\{1-e\left(x\right)\right\}$, where $\:e\left(x\right)$ is the propensity score within that window, and (ii) an outcome-regression term (predicted risk under treatment minus no treatment), then average cross-fitted pseudo-outcomes to obtain the window-specific risk difference for the overlap population. The estimator is consistent if either the propensity or outcome model is correctly specified.

Each window was analyzed separately; within a window, each patient contributes at most one observation. Because patients may contribute to multiple windows and the outcome is terminal, estimates across windows are correlated; we do not combine windows into a single longitudinal inferential model and interpret the pattern over time descriptively.

Both the propensity score model and the outcome model were fitted with a Super Learner (SL) ensemble (GLM, penalized GLM, random forest, shallow gradient boosting) using 5-fold cross-fitting; learner weights were chosen by internal cross-validation (Methods S5). We report effective sample size (ESS) under OWATE, propensity score area under the curve (PS AUC) AUC, Brier score, and covariate balance (median standardised mean difference [SMD]; % SMD < 0.2). Sensitivity analyses additionally estimated a standard AIPW risk difference (i.e., risk difference in the full target population) with stabilised weights and propensity truncation at 0.01 and 0.05.

### Individualised predicted risk differences

We computed individualised risk estimates as the difference in predicted mortality under treatment versus no treatment for each subject using the outcome model.

### Sensitivity analyses

We performed two additional sensitivity analyses excluding patients with shock (noradrenaline > 0.15 µg/kg/min) and excluding patients who received pulse steroids (> 1000 mg PE).

### Software

Analyses were conducted in R. Package details in Methods S5 and code available (https://github.com/Dom-Marshall/Corticosteroids-ARDS).

## Results

A total of 987 subjects were included in the analysis, after excluding subjects without ARDS (Fig. S1). 354 (35.9%) subjects received > 30 mg/3d PE of corticosteroids, of whom 262/354 (74.1%), 161/354 (45.4%), 128/354 (36.2%) received ≥ 150, ≥ 270 and ≥ 390 mg/3d of PE during at least one time window during their ICU stay (Table 1). Corticosteroid recipients had more chronic lung disease and cancer, higher hospital mortality (43.2% vs. 23.4%), vasoactive agent use (34.2% vs. 27.3%), and lower baseline P/F ratios (163 vs. 179 mmHg). Other parameters were similar. Pre-ARDS corticosteroid use was more common among steroid recipients (42.4% vs. 2.6%) and, along with all other prespecified covariates, was included in both the propensity and outcome models. Methylprednisolone was the most frequently prescribed corticosteroid followed by hydrocortisone and prednisolone, details of specific corticosteroid received by day reported in Table S4.

**Table 1:** Baseline variables with subgroups based on corticosteroid dose in Prednisolone Equivalent (PE). For continuous variables median and IQR are presented

Variable	Overall	No corticosteroids	Any Corticosteroids (≥ 30 mg/3d)	≥ 150 mg/3d PE	≥ 270 mg/3d PE	≥ 390 mg/3d PE
*N* (%)	987	612 (62.0)	354 (35.9)	262 (26.5)	161 (16.3)	128 (13.0)
Demographics and Outcomes						
Male, count (%)	618 (62.6)	409 (66.8)	195 (55.1)	143 (54.6)	90 (55.9)	67 (52.3)
Age -yr	62 (51–72)	61 (50–72)	63 (52–72)	62 (52–72)	62 (52–71)	61 (50–69)
ARDS onset from admission – hours (IQR)	8 (2–41)	9 (2–47)	7 (2–32)	7 (2–30)	8 (2–37)	8 (2–49)
Hospital mortality, *n* (%)	302 (30.6)	143 (23.4)	153 (43.2)	117 (44.7)	74 (46.0)	59 (46.1)
ICU mortality, *n* (%)	273 (27.7)	124 (20.3)	144 (40.7)	111 (42.4)	71 (44.1)	56 (43.8)
ICU length of stay - days	12.1 (7.9–18.9)	12.3 (8.3–19.5)	11.7 (7.3–17.8)	11.7 (7.5–17.5)	11.7 (7.5–17.5)	11.8 (7.6–17.5)
Baseline Comorbidities						
Ischaemic heart disease, *n* (%)	140 (14.2)	82 (13.4)	54 (15.3)	38 (14.5)	18 (11.2)	13 (10.2)
Heart failure, *n* (%)	281 (28.5)	165 (27.0)	111 (31.4)	78 (29.8)	48 (29.8)	34 (26.6)
Chronic Lung Disease *n* (%)	343 (34.8)	181 (29.6)	155 (43.8)	118 (45.0)	76 (47.2)	55 (43.0)
Chronic Kidney Disease, *n* (%)	206 (20.9)	129 (21.1)	72 (20.3)	47 (17.9)	26 (16.1)	23 (18.0)
Chronic Liver Disease, *n* (%)	234 (23.7)	143 (23.4)	87 (24.6)	62 (23.7)	30 (18.6)	26 (20.3)
Diabetes Mellitus, *n* (%)	290 (29.4)	179 (29.2)	105 (29.7)	76 (29.0)	45 (28.0)	34 (26.6)
Cancer, *n* (%)	150 (15.2)	58 (9.5)	85 (24.0)	67 (25.6)	46 (28.6)	38 (29.7)
Admission Details						
Direct ARDS	801 (81.2%)	486 (79.4%)	299 (84.5%)	227 (86.6%)	142 (88.2%)	111 (86.7%)
Pneumonia	721 (73.0%)	440 (71.9%)	264 (74.6%)	193 (73.7%)	111 (68.9%)	86 (67.2%)
Surgery	192 (19.5%)	147 (24.0%)	41 (11.6%)	25 (9.5%)	17 (10.6%)	16 (12.5%)
Trauma	79 (8.0%)	69 (11.3%)	10 (2.8%)	7 (2.7%)	3 (1.9%)	3 (2.3%)
Pancreatitis	51 (5.2%)	43 (7.0%)	6 (1.7%)	4 (1.5%)	0 (0.0%)	0 (0.0%)
Corticosteroid administration in 72 h pre-ARDS (PE)						
< 45, *n* (%)	41 (4.2)	6 (1.0)	31 (8.8)	13 (5.0)	3 (1.9)	2 (1.6)
≥ 45 and < 120, *n* (%)	51 (5.2)	8 (1.3)	42 (11.9)	30 (11.5)	14 (8.7)	11 (8.6)
> 120, *n* (%)	81 (8.2)	2 (0.3)	77 (21.8)	68 (26.0)	57 (35.4)	50 (39.1)
Baseline Organ Support						
Inotropes or vasopressors, *n* (%)	297 (30.1)	167 (27.3)	121 (34.2)	87 (33.2)	41 (25.5)	32 (25.0)
Renal replacement therapy, *n* (%)	76 (7.7)	42 (6.9)	31 (8.8)	23 (8.8)	12 (7.5)	9 (7.0)
Positive end expiratory pressure - cmH2O	10 (7–12)	9 (7–12)	10 (7–13)	10 (8–13)	10 (7–13)	10 (8–13)
Driving Pressure - cmH2O	12 (10–15)	12 (10–14)	13 (10–16)	13 (11–16)	14 (11–16)	14 (11–16)
Continuous neuromuscular blockade, *n* (%)	28 (2.8)	14 (2.3)	14 (4.0)	11 (4.2)	6 (3.7)	5 (3.9)
Physiology						
Heart rate – beats/minute	90 (78–102)	89 (78–102)	90 (78–104)	89 (79–104)	89 (78–102)	88 (78–99)
Mean arterial pressure – mmHg	74 (69–80)	74 (69–81)	73 (69–80)	73 (69–80)	73 (70–80)	73 (69–80)
Temperature - C	37.2 (36.8–37.7)	37.3 (36.9–37.8)	37.1 (36.7–37.5)	37.0 (36.7–37.5)	37.0 (36.8–37.4)	37.0 (36.8–37.4)
PaO2/Fio2 ratio - mmHg	172 (133–221)	179 (139–225)	163 (125–208)	156 (120–206)	160 (124–208)	159 (124–206)
Partial pressure CO2 – mmHg	43 (38–49)	42 (38–47)	44 (38–51)	44 (38–52)	48 (40–53)	45 (40-54.4)
pH	7.36 (7.30–7.40)	7.37 (7.31–7.41)	7.34 (7.28–7.39)	7.34 (7.27–7.39)	7.34 (7.28–7.40)	7.34 (7.28–7.40)
Lactate - mmol/L	1.7 (1.2–2.6)	1.7 (1.2–2.5)	1.7 (1.2–2.9)	1.7 (1.1–2.9)	1.4 (1.0-2.3)	1.4 (1.0-2.3)
Creatinine – mg/dL	1.20 (0.78–2.02)	1.20 (0.77-2.00)	1.23 (0.80–2.03)	1.21 (0.80–1.99)	1.06 (0.76–1.77)	1.03 (0.75–1.75)
Haemoglobin – g/dL	9.9 (8.7–11.3)	10.0 (8.8–11.4)	9.7 (8.7–11.2)	9.7 (8.7–11.3)	9.57 (8.6–11.3)	9.51 (8.6–11.3)
White cell count - cells ×10^9^/L	12.2 (8.9–17.1)	12.0 (9.0-16.8)	12.9 (8.5–17.7)	12.2 (8.2–17.8)	11.6 (8.5–16.4)	11.2 (8.2–15.8)

### Model performance and overlap

**Fig. 1:**
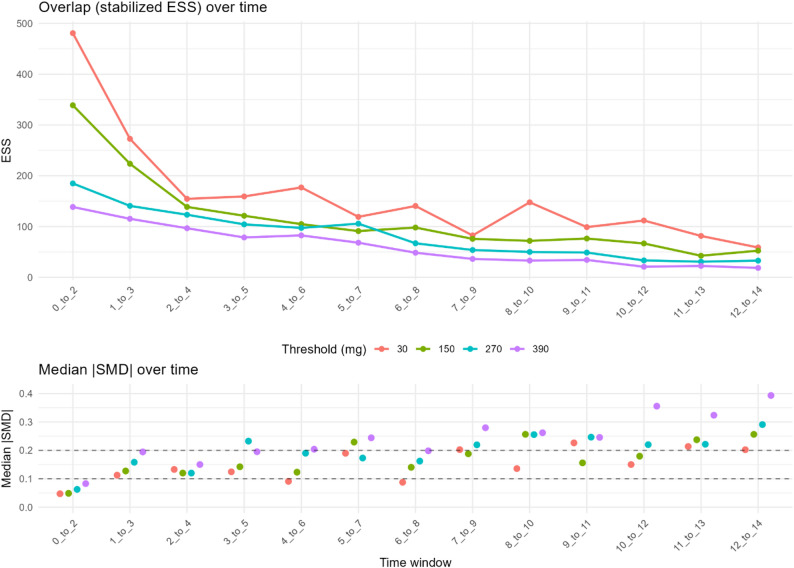
Overlap and covariate balance across sliding time windows. Top panel: effective sample size (ESS). Lines show the effective sample size per 3-day window for each prednisolone-equivalent (PE) dose threshold (30, 150, 270, 390 mg/3d), color-coded by threshold. Windows are labelled 0–2, 1–3, …, 12–14 days from ARDS onset. ESS is computed from the overlap weights used in the primary analysis (OWRD); higher ESS indicates better covariate overlap between treated and untreated within that window. Bottom panel: median standardized mean difference (SMD). Dots show the median absolute standardized mean difference across covariates after applying the same OWRD weights. Dashed reference lines mark SMD = 0.10 and 0.20; lower values indicate better balanceAll estimates are pooled across imputations

We used 25 covariates for the propensity and outcome models; pruning to this parsimonious set did not materially change model performance compared with the larger candidate list (Table S1). Propensity score performance was consistent across windows, demonstrating good discrimination and low calibration error (Figs. S3, S4; Table S1). However, covariate overlap decreased over time and at higher corticosteroid doses (Fig. 1). For example, the effective sample size at days 0–2 dropped from 481 (at the ≥ 30 mg/3d threshold) to 139 (at ≥ 390 mg/3d). By days 12–14, these effective sample sizes declined to 59 and 19, for ≥ 30 mg/3d and ≥ 390 mg/3d respectively (Table S1). Covariate balance followed a similar pattern (Fig. 1): the proportion of covariates with an absolute standardized mean difference (SMD) < 0.20 was 94% in early windows (days 0–2, ≥ 30 mg) but fell to 35% in late, high-dose windows (days 12–14, ≥ 390 mg).

### Adjusted associations between corticosteroid exposure and hospital mortality

**Fig. 2:**
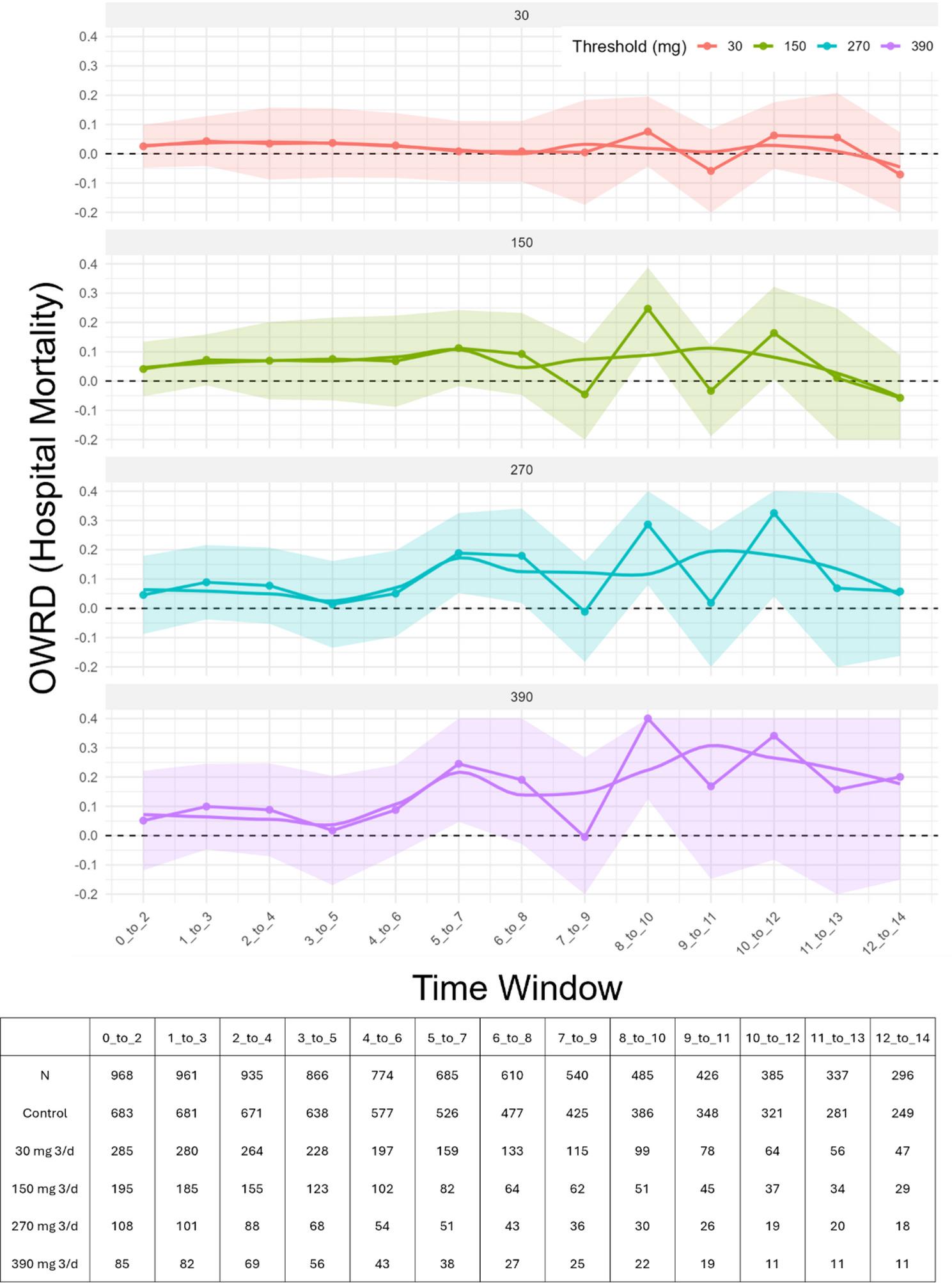
Overlap-Weighted AIPW Risk Difference (OWRD) estimates of hospital-mortality risk difference across overlapping 3-day windows, faceted by corticosteroid dose threshold (prednisolone-equivalent: ≥30, ≥150, ≥270, ≥390 mg/3d). Points show window-specific OWRD estimates; shaded ribbons denote 95% CIs; the dashed horizontal line marks risk difference = 0. A LOESS curve is overlaid for visual trend only. Windows are defined relative to ARDS onset (0–2, 1–3, …, 12–14 days); exposure is assessed within each window and follow-up begins at the window’s end. All estimates are pooled across imputations. The risk table beneath the panels gives per-window counts (*N*, treated, control) corresponding to the plotted estimates

At the lowest threshold (≥ 30 mg/3d), window-specific adjusted risk differences were close to zero throughout days 0–14 (Figure 2, Table 2). For example, at days 0–2, the overlap-weighted AIPW risk difference (OWRD) was 0.03 (95% CI − 0.05 to 0.10). This indicates that, in the overlap-weighted population, corticosteroid exposure ≥ 30 mg/3d was associated with a non-significant 3% absolute difference in hospital mortality. Across the ICU stay for this threshold, the highest OWRD was 0.08 (95% CI − 0.04 to 0.20) at days 8–10, and the lowest was − 0.07 (− 0.22 to 0.07) at days 12–14.

**Table 2:** Window-specific Overlap-weighted AIPW Risk Difference (OWRD) for hospital mortality, shown as risk difference with 95% confidence interval. Columns correspond to cumulative prednisolone-equivalent dose thresholds over 3 days (≥ 30, ≥ 150, ≥270, ≥ 390 mg/3d). Rows are consecutive 3-day windows ordered chronologically. Positive OWRD indicates higher mortality risk associated with corticosteroids

Window	Corticosteroid Dose Threshold mg/3d Prednisolone Equivalent			
	≥ 30	≥ 150	≥ 270	≥ 390
0–2	0.03 (-0.05-0.10)	0.04 (-0.05-0.13)	0.05 (-0.09-0.18)	0.05 (-0.12-0.22)
1–3	0.04 (-0.04-0.13)	0.07 (-0.02-0.16)	0.09 (-0.04-0.22)	0.10 (-0.05-0.25)
2–4	0.04 (-0.09-0.16)	0.07 (-0.06-0.20)	0.08 (-0.05-0.21)	0.09 (-0.07-0.25)
3–5	0.04 (-0.08-0.16)	0.08 (-0.07-0.22)	0.01 (-0.14-0.16)	0.02 (-0.17 -0.20)
4–6	0.03 (-0.08-0.14)	0.07 (-0.09-0.22)	0.05 (-0.10-0.20)	0.09 (-0.07-0.24)
5–7	0.01 (-0.10-0.11)	0.11 (-0.02-0.24)	0.19 (0.05–0.33)	0.25 (0.05–0.44)
6–8	0.01 (-0.10-0.11)	0.09 (-0.05-0.23)	0.18 (0.02–0.34)	0.19 (-0.03-0.41)
7–9	0.01 (-0.17-0.18)	-0.05 (-0.22-0.13)	-0.01 (-0.18-0.16)	-0.01 (-0.28-0.27)
8–10	0.08 (-0.04-0.20)	0.25 (0.11–0.39)	0.29 (0.08–0.49)	0.41 (0.12–0.69)
9–11	-0.06 (-0.20-0.08)	-0.03 (-0.19-0.12)	0.02 (-0.23-0.26)	0.17 (-0.15-0.48)
10–12	0.06 (-0.05-0.18)	0.16 (0.01–0.32)	0.33 (0.04–0.61)	0.34 (-0.08-0.76)
11–13	0.06 (-0.10-0.21)	0.01 (-0.23-0.25)	0.07 (-0.26-0.39)	0.16 (-0.21-0.52)
12–14	-0.07 (-0.22-0.07)	-0.06 (-0.20-0.09)	0.06 (-0.16-0.28)	0.20 (-0.15-0.55)

At higher thresholds, we observed a pattern of increasing OWRDs (higher mortality) in mid-to-late windows, though precision decreased significantly. At ≥ 150 mg/3d, early windows were modest (e.g., 0.04, − 0.05 to 0.13 at 0–2 d), while larger positive OWRD occurred in the middle windows: 0.25 (0.11 to 0.39) at 8–10 d (largest), and 0.16 (0.01 to 0.32) at 10–12 days; by 12–14 days the estimate was − 0.06 (− 0.20 to 0.09).

At ≥ 270 mg/3d, OWRDs were again small early (0.05, − 0.09 to 0.18 at 0–2 days) and larger mid-course, with notable windows 5–7 days: 0.19 (0.05 to 0.33), 6–8 d: 0.18 (0.02 to 0.34), 8–10 days: 0.29 (0.08 to 0.49), and the largest at 10–12 days: 0.33 (0.04 to 0.61).

At ≥ 390 mg/3d, early windows estimated OWRDs were small (0.05, − 0.12 to 0.22 at 0–2 days), with larger mid-course estimates: 5–7 days: 0.25 (0.05 to 0.44), and the largest at 8–10 days: 0.41 (0.12 to 0.69). Later windows had wider intervals with fewer treated patients (e.g., 10–12 days: 0.34, − 0.08 to 0.76; 12–14 days: 0.20, − 0.15 to 0.55).

### Individualized predicted risk differences

Distributions of individualized predicted risk differences were consistent with the aggregate findings. They were narrow and centered near zero in early windows across all dose thresholds. In later windows, the distributions became progressively wider, showing greater dispersion and a shift toward higher predicted mortality risk at higher dose thresholds (Fig. S5).

### Sensitivity analyses

Alternative weighting using truncated risk differences (propensity truncation at 0.01 and 0.05) produced window-specific risk-difference series that tracked the above results (Fig. S6). Similarly, excluding patients in shock and/or those receiving pulse-dose corticosteroids yielded findings similar to the primary analysis, with wider confidence intervals and smaller effective sample sizes in later, higher-dose windows (Fig. S7, Table S3).

## Discussion

In this retrospective sliding-window analysis of time-varying corticosteroid exposure in ARDS, we used overlap-weighted AIPW estimate to examine how associations between cumulative corticosteroid dose and hospital mortality vary with time from ARDS onset. Overall, corticosteroid treatment showed minimal time-varying association with mortality during the first two weeks. We observed greater variation over time in the higher doses of corticosteroids with a signal of higher hospital mortality with higher doses of corticosteroids in mid and late time windows. These findings must be interpreted with caution given the reduction in precision and effective sample size later in the ICU stay.

Interpretation of the late/high-dose associations requires particular nuance. In routine practice, escalation to high cumulative doses later in ICU stay often reflects refractory disease, failure of other therapies, or end-of-life decision contexts, factors that may not be fully captured by structured EHR variables. Residual confounding by clinical trajectory therefore remains a plausible explanation for the observed higher mortality in late windows at ≥ 150–390 mg/3d thresholds. These estimates are further limited by the small effective sample sizes at higher doses and later time points.

Our findings of a potentially time varying association of corticosteroids are consistent with results of randomised trials where early use has been associated with benefit but later use with harm [7, 12, 15]. It is biologically plausible that corticosteroids have differential effects if given at different times during the disease process, perhaps reflecting distinct phases of ARDS. Early administration may attenuate inflammation and reduce alveolar damage [16, 17], potentially linked to improved NF-κB/GRα activity. However, late administration may be less beneficial or harmful due to complications (neuromyopathy, hyperglycaemia and gastrointestinal bleeding) and irreversible tissue damage from fibroproliferation [7, 18, 19]. 

Recent multicenter observational work in sepsis using trajectory-stratified causal inference methods supports this complexity, reporting that corticosteroids may contribute to harm in patients whose clinical trajectories suggest rapid improvement [20]. Similarly, studies looking at biological sub-phenotypes of critical illness including ARDS have identified groups that may come to harm with these drugs [21, 22] highlighting the possibility of biologically driven adverse effects rather than simply confounding by indication. However, as the majority of this work has focused on cross sectional, single time point data and our findings support the need for longitudinal subphenotyping to further understand disease progression and treatment responsiveness over time [23, 24]. 

Our results also align with other observational studies associating corticosteroids with higher mortality [25–28]. The discrepancy with randomised trials [14, 29], may arise because trials enrol selected populations receiving early intervention [30]. By contrast, the MIMIC-IV database encompasses a broader, more heterogenous group of ICU patients who may not have met trial criteria and many of whom received corticosteroids for indications beyond ARDS (e.g., septic shock, COPD exacerbations, or other inflammatory conditions). Further, higher doses used later may reflect ‘last resort’ use in critically ill patients expected to have poor outcomes [31], however, our sensitivity analysis excluding pulse-dose corticosteroids suggests this practice alone does not fully explain the findings.

Despite the strengths of this study, including the use of a large, high-granularity database and rigorous doubly robust adjustment (AIPW), it has limitations. First, even with propensity adjustment, unobserved confounders may remain. Key unmeasured variables, such as the specific clinician rationale for corticosteroid initiation (e.g., “salvage” therapy), might bias our estimates toward a harmful association. Second, modeling exposure via cumulative doses and discrete thresholds within overlapping 3-day windows serves as a pragmatic parameterization. Because the majority of these exposures represent therapy continuations rather than *de novo* prescriptions, utilizing alternative window lengths or continuous dose specifications might have yielded different associative patterns. Third, patients contribute to multiple overlapping windows with a single terminal outcome; therefore, the series of window-specific estimates are correlated and should be interpreted descriptively rather than as independent hypothesis tests. Finally, the cohort represents data from a single center over an extended era, introducing potential practice drift, and encompasses heterogeneous ARDS etiologies (e.g., sepsis vs. trauma) that may shape treatment decisions in ways not fully captured by our models. Although expert chart review was used to exclude patients presenting with chronic lung disease exacerbations rather than ARDS, the high baseline prevalence of chronic lung disease in our cohort may still introduce residual confounding.

We observed no clear association between early, lower cumulative corticosteroid exposure and hospital mortality. In contrast, higher cumulative doses later in the ICU stay were associated with higher mortality, though these estimates are difficult to interpret due to limited overlap and likely residual confounding by clinical trajectory. These findings are hypothesis-generating and support prospective studies explicitly testing the timing and dose of corticosteroids in ARDS (Fig. 2).

## Supplementary Information

Supplementary Material 1.

Supplementary Material 2.

## Data Availability

The MIMIC-IV database is publicly available via [https://physionet.org/content/mimiciv/1.0/] for researchers who have signed the data user agreement and completed the required training. With permission from the Physionet team we include a list of stay\_ids for our cohort in the supplementary materials. Additional disaggregated cohort details are available on request to the corresponding author to appropriately credential Physionet users.
