# Supplementary material for: Time-varying associations between corticosteroid dose and hospital mortality in ARDS: a sliding-window analysis of MIMIC-IV
Source: BMC Pulm Med. 2026 Mar 26;26:209. doi: 10.1186/s12890-026-04251-w (PMC13141438; doi:10.1186/s12890-026-04251-w)
Supplement: Supplementary file 1 — Supplementary Material 1. [file 12890_2026_4251_MOESM1_ESM.docx]

# STROBE Checklist (Cohort/Observational Study)

| Item | Recommendation | Reported? | Location in manuscript | Notes |
| --- | --- | --- | --- | --- |
| 1a | Title: Indicate the study design with a commonly used term in the title or the abstract. | Yes | Title; Abstract |  |
| 1b | Abstract: Provide an informative and balanced summary of what was done and what was found. | Yes | Abstract |  |
| 2 | Background/rationale: Explain the scientific background and rationale for the investigation being reported. | Yes | Introduction |  |
| 3 | Objectives: State specific objectives, including any prespecified hypotheses. | Yes | Introduction (last paragraph) |  |
| 4 | Study design: Present key elements of study design early in the paper. | Yes | Methods (first paragraphs) |  |
| 5 | Setting: Describe the setting, locations, and relevant dates, including periods of recruitment, exposure, follow-up, and data collection. | Yes | Methods – Data Source (MIMIC-IV 2008–2019); follow-up to discharge |  |
| 6a | Participants: Give the eligibility criteria, and the sources and methods of selection of participants; describe methods of follow-up. | Yes | Methods – Subject Selection; Supplementary Methods S1; Fig S1 |  |
| 6b | For matched studies, give matching criteria and number of exposed and unexposed. | N/A | Not a matched study | Not applicable to this study design. |
| 7 | Variables: Clearly define all outcomes, exposures, predictors, potential confounders, and effect modifiers; give diagnostic criteria, if applicable. | Yes | Methods – Variables (dose thresholds, covariates, Berlin criteria); Supp. Methods S3 |  |
| 8* | Data sources/measurement: For each variable of interest, give sources of data and details of methods of assessment. | Yes | Methods – Data Source; Pre-processing; Supp. Methods S2–S5 |  |
| 9 | Bias: Describe any efforts to address potential sources of bias. | Yes | Methods – Design (target-trial emulation, OWATE/AIPW); Discussion – Limitations |  |
| 10 | Study size: Explain how the study size was arrived at. | Partial | Methods – Subject Selection (all eligible ARDS in MIMIC-IV; no formal power calculation) |  |
| 11 | Quantitative variables: Explain how quantitative variables were handled in the analyses. If applicable, describe which groupings were chosen and why. | Yes | Methods – Sliding windows; dose thresholds; covariate summaries |  |
| 12a | Statistical methods: Describe all statistical methods, including those used to control for confounding. | Yes | Methods – AIPW/OWATE; Super Learner; overlap weights |  |
| 12b | Describe any methods used to examine subgroups and interactions. | Yes | Methods – Non-shocked and No steroid pulse group defined. |  |
| 12c | Explain how missing data were addressed. | Yes | Supplementary Methods S2 (MICE with pmm; pooling by Rubin’s rules) |  |
| 12d | Cohort study—If applicable, explain how loss to follow-up was addressed. | Yes | Methods – Follow-up to discharge post-window; hospital mortality outcome |  |
| 12e | Describe any sensitivity analyses. | Yes | Methods – Sensitivity analyses; Supp. Figs S6–S7; Table S3 |  |
| 13a | Participants: Report numbers of individuals at each stage of study. | Yes | Results – Cohort counts; Supp. Fig S1; Tables |  |
| 13b | Give reasons for non-participation at each stage. | Partial | Supp. Fig S1/Methods S1 describe exclusions; reasons summarized |  |
| 13c | Consider use of a flow diagram. | Yes | Supplementary Figure S1 (CONSORT-style diagram) |  |
| 14a | Descriptive data: Give characteristics of study participants and information on exposures and potential confounders. | Yes | Table 1 |  |
| 14b | Indicate number of participants with missing data for each variable of interest. | Partial | Missingness approach described (Supp. S2); variable-level missingness not tabulated |  |
| 14c | Summarise follow-up time (e.g., average and total amount). | Partial | Follow-up until hospital discharge described; no person-time summary |  |
| 15 | Outcome data: Report numbers of outcome events or summary measures over time. | Yes | Results – Mortality counts; Table 2; Figures |  |
| 16a | Main results: Give unadjusted estimates and, if applicable, confounder-adjusted estimates and their precision. | Partial | Adjusted OWATE risk differences with 95% CI reported; unadjusted not presented |  |
| 16b | Report category boundaries when continuous variables were categorized. | Yes | Methods – Dose thresholds (≥30, ≥150, ≥270, ≥390 mg/3d) |  |
| 16c | If relevant, consider translating estimates of relative risk into absolute risk for a meaningful time period. | Yes | Risk differences (absolute) presented per window |  |
| 17 | Other analyses: Report other analyses done—e.g., analyses of subgroups and interactions, and sensitivity analyses. | Yes | Results – Sensitivity analyses; ITE distributions |  |
| 18 | Key results: Summarise key results with reference to study objectives. | Yes | Discussion – first paragraphs |  |
| 19 | Limitations: Discuss limitations of the study, taking into account sources of potential bias or imprecision. Discuss both direction and magnitude of any potential bias. | Yes | Discussion – Limitations |  |
| 20 | Interpretation: Give a cautious overall interpretation of results considering objectives, limitations, multiplicity of analyses, results from similar studies, and other relevant evidence. | Yes | Discussion |  |
| 21 | Generalisability: Discuss the generalisability (external validity) of the study results. | Yes | Discussion – Limitations/Generalisability |  |
| 22 | Funding: Give the source of funding and the role of the funders. | Yes | Declarations – Funding and acknowledgements |  |

# RECORD Checklist (Extension for Routinely Collected Data)

| Item | Recommendation | Reported? | Location in manuscript | Notes |
| --- | --- | --- | --- | --- |
| RECORD 1.1 | Type of data: Specify type of data used (e.g., EHR, claims); include database name and geographic/temporal coverage. | Yes | Methods – Data Source (MIMIC-IV; BIDMC; 2008–2019) |  |
| RECORD 1.2 | Data linkage: If applicable, indicate whether the study involved data linkage across two or more databases. | N/A | No external linkage (single database) | Not applicable to this study. |
| RECORD 6.1 | Study population selection: Methods of selecting the study population using codes/algorithms; validation references if available. | Partial | Methods S1 (algorithm using P/F and PEEP; Berlin criteria by chart review); Supp. Fig S1 |  |
| RECORD 6.2 | Validation study: Reference validation studies of codes/algorithms used to select the population, or describe validation. | Yes | Supplementary Methods S1 (expert chart review; κ=0.76) |  |
| RECORD 7.1 | Variables: Provide codes and algorithms used to classify exposures, outcomes, confounders; include code lists or explain where available. | Yes | Methods – Dose conversion (NICE eq. table); Supp. S3; full code on GitHub |  |
| RECORD 12.1 | Data access and cleaning methods: Describe how data were accessed and cleaned. | Yes | Methods – SQL BigQuery extraction; pre-processing; imputation (Supp. S2) |  |
| RECORD 12.2 | Data linkage methods and quality evaluation. | N/A | No linkage performed | Not applicable to this study. |
| RECORD 13.1 | Participants: Describe the selection of persons included in the study using a flow diagram. | Yes | Supplementary Figure S1 |  |
| RECORD 19.1 | Limitations of using routinely collected data (misclassification, unmeasured confounding, missing data, changes in coding). | Yes | Discussion – Limitations |  |
| RECORD 22.1 | Data sharing: Provide information on how to access supplemental materials, protocol, raw data, or programming code. | Yes | Data availability (PhysioNet MIMIC-IV); Code: GitHub repo |  |

.
