# Supplementary material for: Time-varying associations between corticosteroid dose and hospital mortality in ARDS: a sliding-window analysis of MIMIC-IV
Source: BMC Pulm Med. 2026 Mar 26;26:209. doi: 10.1186/s12890-026-04251-w (PMC13141438; doi:10.1186/s12890-026-04251-w)
Supplement: Supplementary file 2 — Supplementary Material 2. [file 12890_2026_4251_MOESM2_ESM.docx]

**Supplementary Materials: Time-varying associations between corticosteroid dose and hospital mortality in ARDS: a sliding-window analysis of MIMIC‑IV**

Dominic C Marshall^1,2*^, Matthieu Komorowski^1^, Brijesh V Patel^1^, David B Antcliffe^1^, Sonali Parbhoo^3^

1. Division of Anaesthetics, Pain Medicine and Intensive care, Department of Surgery and Cancer, Imperial College London, United Kingdom
2. Cleveland Clinic London, London, United Kingdom
3. School of Electrical and Electronic Engineering, Imperial College London, London, United Kingdom

*Corresponding author: dominic.marshall12@imperial.ac.uk

**Index**

**Additional Methods**

Methods S1 - Cohort selection – P3

Methods S2 - Missing data and imputation – P4

Methods S3 - Covariate curation, pruning, and diagnostics – P5

Methods S4. AIPW and OWRD estimation and weighting details – P6

Methods S5. Super Learner models and software – P7

**Supplementary figures**

Figure S1 – Consort diagram for ARDS selection – P8

Figure S2 – Direct Acyclic Graph – P9

Figure S3 – Propensity model performance – P10

Figure S4 – Propensity model calibration – P11

Figure S5 – Individualized predicted risk differences – P12

Figure S6 – Sensitivity to overlap – AIPW RD vs OWATE – P13

Figure S7 – Sensitivity analyses (Pulse corticosteroids and shock – P14

**Supplementary tables**

Table S1 – Model covariates – P15

Table S2 – Full OWRD results with overlap-weighted diagnostics – P16

Table S3 – Full results for sensitivity analyses – P17

Table S3 – Corticosteroid prescription information P19

**Stay_ids for ARDS patient cohort – P20**

**Additional methods**

**Methods S1 - Cohort selection Identification of Potential ARDS Cohort**

1. Initial Screening
   - Patients were screened if, at any point during their ICU stay, they were mechanically ventilated and had a partial pressure of arterial oxygen to fraction of inspired oxygen (P/F) ratio below 300 mmHg while on a positive end-expiratory pressure (PEEP) of 5 cmH₂O or higher.
2. Averaging Measurements
   - For each qualifying day (and the subsequent two days), the average P/F ratio and PEEP were calculated. These rolling averages were used to form two descriptive subgroups (“24-hour” and “48-hour” cohorts).
3. Final Inclusion Criteria
   - Patients entered the “Final Cohort” cohort if they met one of the following:
     - Persistent Criteria Over 72 Hours: An average P/F ratio below 300 mmHg and an average PEEP of at least 5 cmH₂O for three consecutive days.
     - Persistent Criteria with Mortality on Day Three: Meeting the same P/F and PEEP thresholds for two consecutive days, followed by death on the third day.
   - The onset of a potential ARDS episode was the first day these criteria were satisfied.
4. Expert review
   - Patients underwent review of clinical information and were identified as ARDS if they met Berlin Criteria.

Rationale

- Clinical Relevance: Using a three-day requirement enhanced diagnostic specificity by minimizing the inclusion of short-lived hypoxemia. Previous research suggests that persistent hypoxemia is more likely to represent “true” ARDS.
- Data Adequacy: A three-day window also ensured the availability of adequate clinical information (e.g., imaging, echocardiography, and clinical documentation) for expert evaluation.

Expert Review

- A random sample of 2,000 patients from the 72-hour cohort (those meeting the above criteria) underwent expert review. Discharge summaries, radiology reports, echocardiogram findings, and clinical notes were examined to categorize each patient as “ARDS,” “not ARDS,” or “possible ARDS.”
- Of the 2,000 patients, 987 were identified as ARDS. A subset of 100 random cases was independently reviewed by a second assessor, yielding an agreement rate of 86% (Κ = 0.76). Disagreements occurred only between “possible ARDS” and the other two classifications with disagreements resolved with pre-specified rules.

Note:

The relatively low ARDS incidence reflects MIMIC-IV’s high surgical and cardiac ICU patient composition, populations less frequently meeting stringent ARDS diagnostic criteria

**Methods S2. Missing data and imputation**

Carry-forward step. Non-ABG laboratory values were forward-filled with last-observation-carried-forward (maximum gap 48 hours).

Multiple imputation: Remaining missing data were imputed with multiple imputation by chained equations (MICE) using predictive mean matching (pmm). Analyses were performed separately in each imputed dataset and pooled using Rubin’s rules.

**Methods S3. Covariate curation, pruning, and diagnostics**

A priori set. Guided by a prespecified DAG and clinical expertise, we assembled ~280 candidate covariates, including:

- Static features (demographics, comorbidities, ARDS aetiology),
- History indicators (pre-ARDS steroids and prior-window steroid use),
- Window-level summaries of dynamic variables (e.g., mean, min, max, range, variance; day-specific values for ventilator/gas-exchange).

De-duplication & pruning. To avoid redundancy and over-adjustment, we collapsed overlapping summaries within each variable family (e.g., retained mean or range, not both) using cross-validated importance from the propensity score (PS) and outcome (μ) Super Learners. We then iteratively pruned, re-fitting PS and μ at each step, and stopped when further removal degraded PS AUROC/Brier or weighted balance (median |SMD|; %|SMD|<0.10) or worsened μ-model Brier/log-loss.

Final sets. The PS model used ~25 covariates per window with history terms forced-in. The μ-model used a partially overlapping set.

Diagnostics. Per window we report PS AUROC, Brier score, median |SMD|, %|SMD|<0.1, effective sample size (ESS), and propensity truncation rates (where applicable). Full variable lists (PS and μ, by window) are provided in Supplementary Data File.

**Methods S4. AIPW and OWRD estimation and weighting details**

*Estimands*

The primary estimand per window was the overlap-weighted AIPW estimated risk difference (OWRD) on the risk-difference scale (average association among patients with good covariate overlap). Sensitivity analyses reported the standard AIPW RD with propensity truncation.

*AIPW Estimation Procedure*

The augmented inverse probability weighting (AIPW) estimator was used to estimate the adjusted association between corticosteroids and outcomes. AIPW combines:

1. Inverse Probability Weighting (IPW): Reweights the observed outcomes by the inverse probability of treatment.
2. Outcome Regression: Incorporates a model of the outcome to adjust for residual confounding and correct potential bias in the IPW estimates.

Formally, the AIPW estimator is constructed by summing (i) the IPW term for each subject and (ii) the outcome regression term for that subject. The final ‘aggregated difference’ across the cohort (averaging the difference in predicted outcomes under treatment vs. no treatment) is the ATE.

*OWRD implementation*

OWRD was computed by weighting the AIPW influence function by $h\left( X \right)=e\left( X \right) \left\{ 1-e\left( X \right) \right\}$, where $e\left( X \right)$ is the estimated propensity score.

- Weight: $h(X)=e^(X)\{1-e^(X)\}.$
- AIPW: $\varphi i=m^1(Xi)-m^0(Xi)+Ai(Yi-m^1(Xi))/e^(Xi)-(1-Ai)(Yi-m^0(Xi))/(1-e^(Xi))$
- Estimator: $\hat{\tau}OWATE=\sum hi\varphi i/\sum h$

Notation as in the main text (Treatment *A¸* outcome *Y,* covariates *X*, propensity $e$ and outcome regression $m_{a}$).

Stabilization and truncation. For ATE sensitivity analyses, we used stabilized IP weights and propensity-score truncation at 0.01 and 0.05. We report truncation proportions per window in the diagnostics.

Uncertainty. Standard errors used the empirical variance of the (weighted) influence function with normal approximations; estimates were pooled across imputations using Rubin’s rules.

Exposure coding in windows. Within each 3-day window, exposure was defined as meeting a dose threshold (≥30, ≥150, ≥270, ≥390 mg prednisolone-equivalent). Windows with 0<dose<threshold were excluded for that window to ensure a stable contrast.

**Methods S5. Super Learner models and software**

Both the propensity score and outcome models were fit using a Super Learner (SL) ensemble with a prespecified library:

- generalized linear models (glm),
- penalized GLMs (glmnet),
- random forests (randomForest),
- shallow gradient boosting (xgboost).

Training scheme. We used 5-fold cross-fitting to obtain out-of-fold predictions for AIPW; SL learner weights were selected by internal cross-validation within each window. Hyper-parameters, cross-validation settings, and seeds are specified in the project code.

Software
Analyses were conducted in R. Core packages included mice (MICE with pmm), SuperLearner, xgboost, randomForest, glmnet, and data-wrangling/plotting dependencies. Exact package versions and scripts are provided with the project code (https://github.com/Dom-Marshall/Corticosteroids-ARDS).


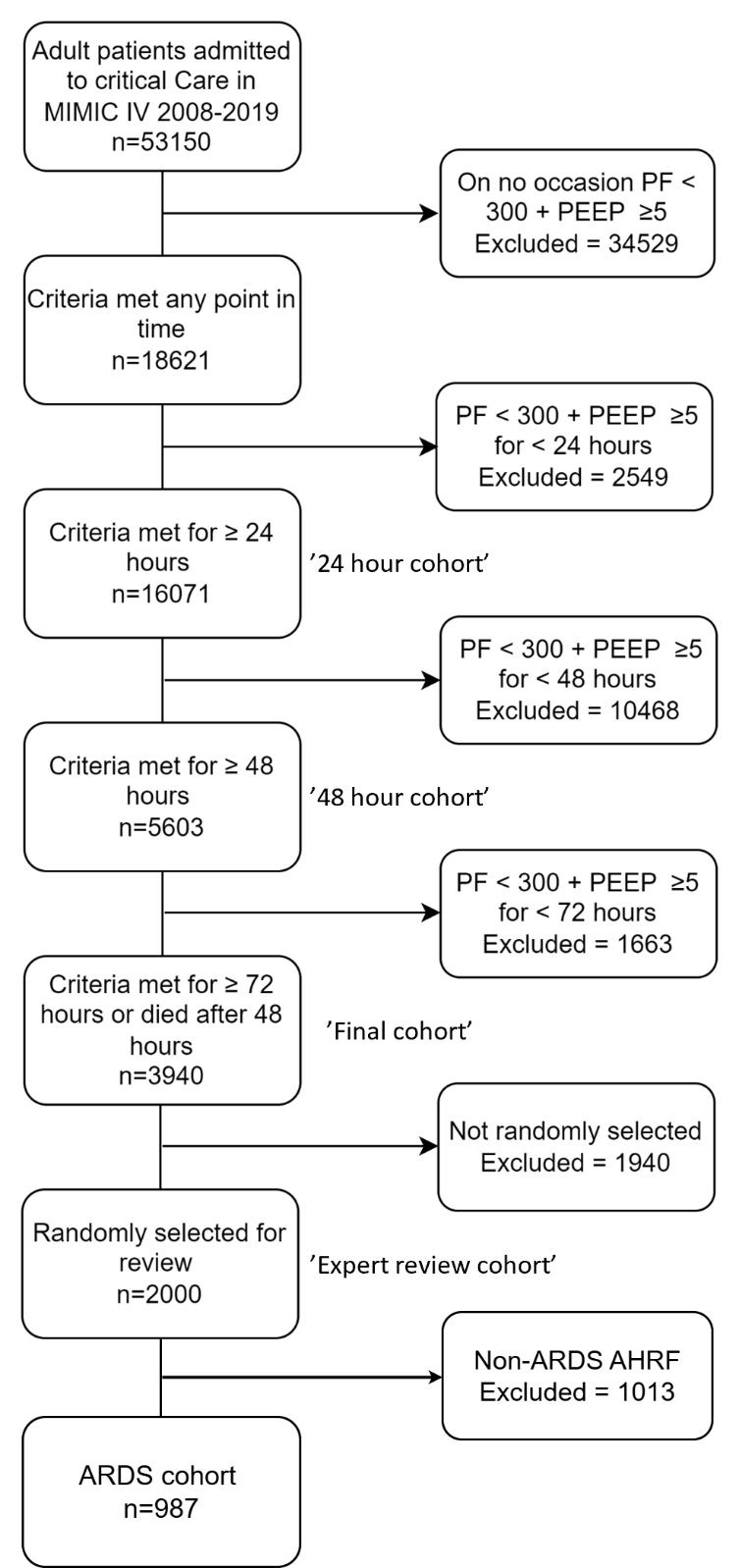


**Figure S1:** Consort diagram demonstrating selection process for ARDS cohort used in AIPW analysis. AHRF – Acute hypoxaemic respiratory failure

**Figure S2:** Directed acyclic graph depicting the assumed relationships among baseline severity, evolving clinical status, corticosteroid exposure within a 3-day window, and hospital mortality. The minimal sufficient adjustment set informed covariate selection.


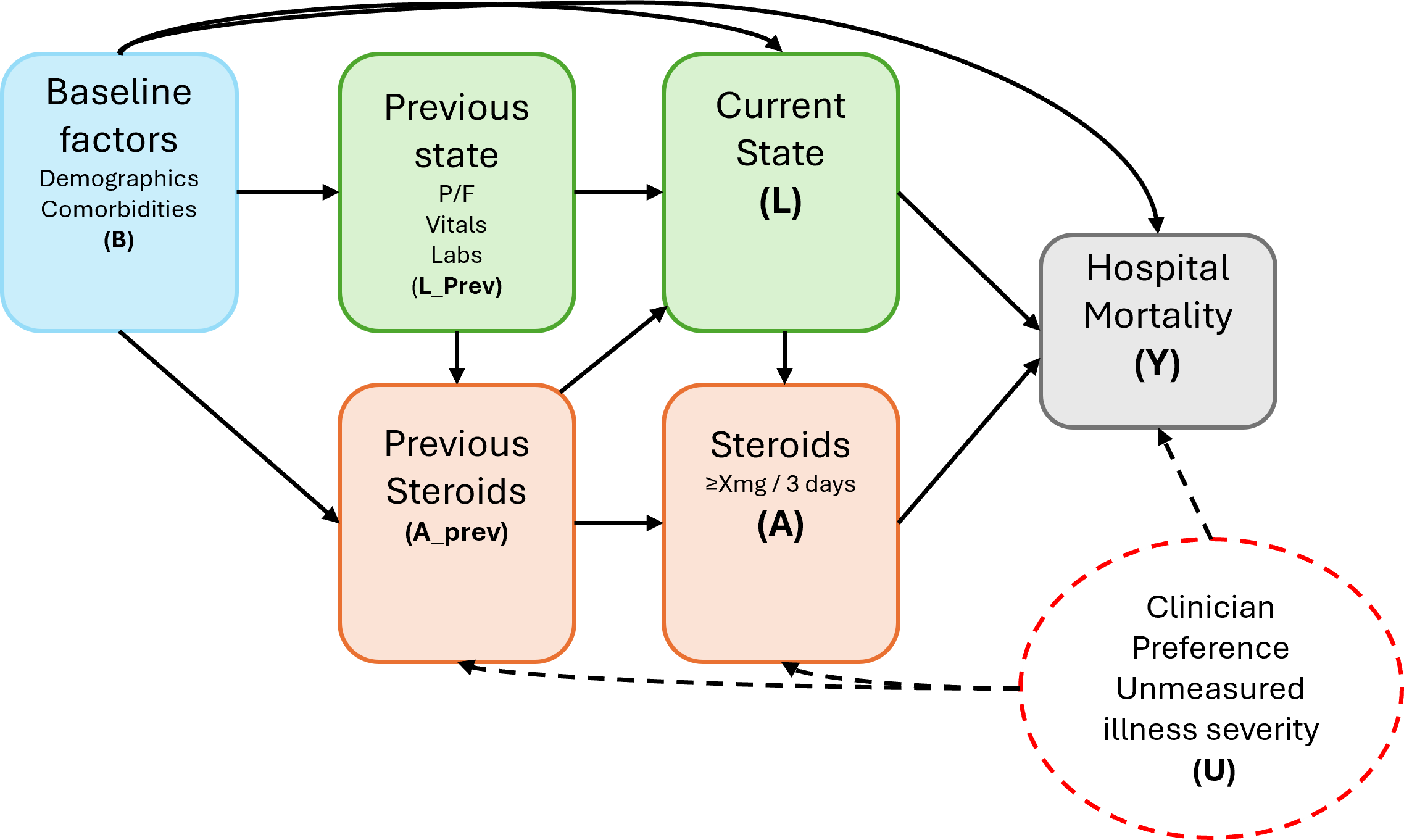


**Figure S3:** Propensity model performance across sliding windows
Lines show discrimination (AUC, top) and calibration error (Brier score, bottom) for the propensity score model within each 3-day window (0–2, 1–3, …, 12–14 days), stratified by prednisolone-equivalent (PE) thresholds (30, 150, 270, 390 mg/3d). Higher AUC indicates better discrimination of treated vs untreated; lower Brier indicates better overall calibration. Results reflect the primary cohort (including shock and pulse-dose patients) and are pooled across imputations.


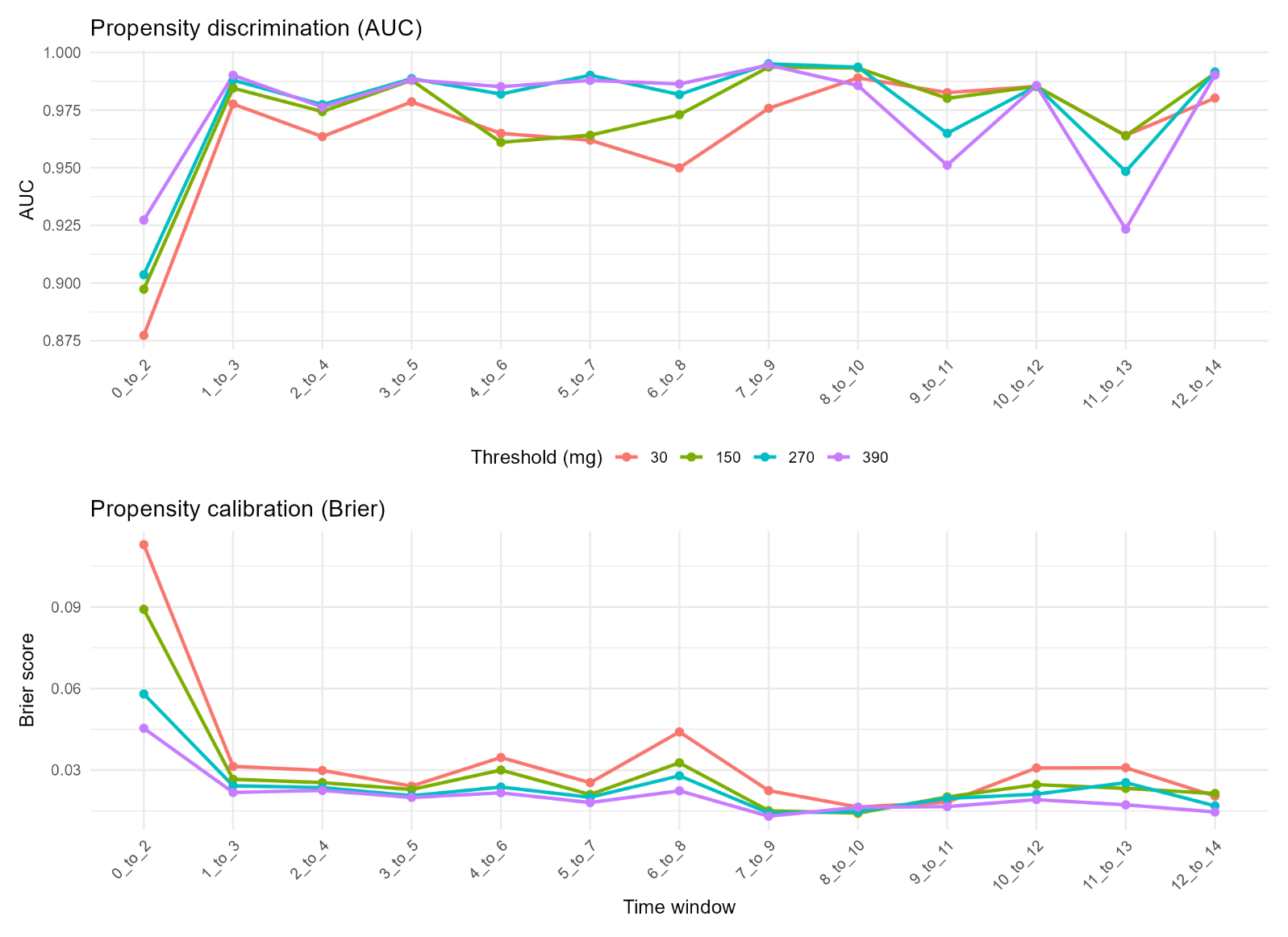


**Figure S4.** Decile calibration of the propensity score by window and dose threshold
Four panels (30, 150, 270, 390 mg/3d). Within each panel, points show observed treatment probability vs mean predicted probability by decile, separately for each 3-day window (faceted). The 45° dashed line denotes perfect calibration; point size is proportional to bin size. Analyses use the primary cohort and are pooled across imputations.


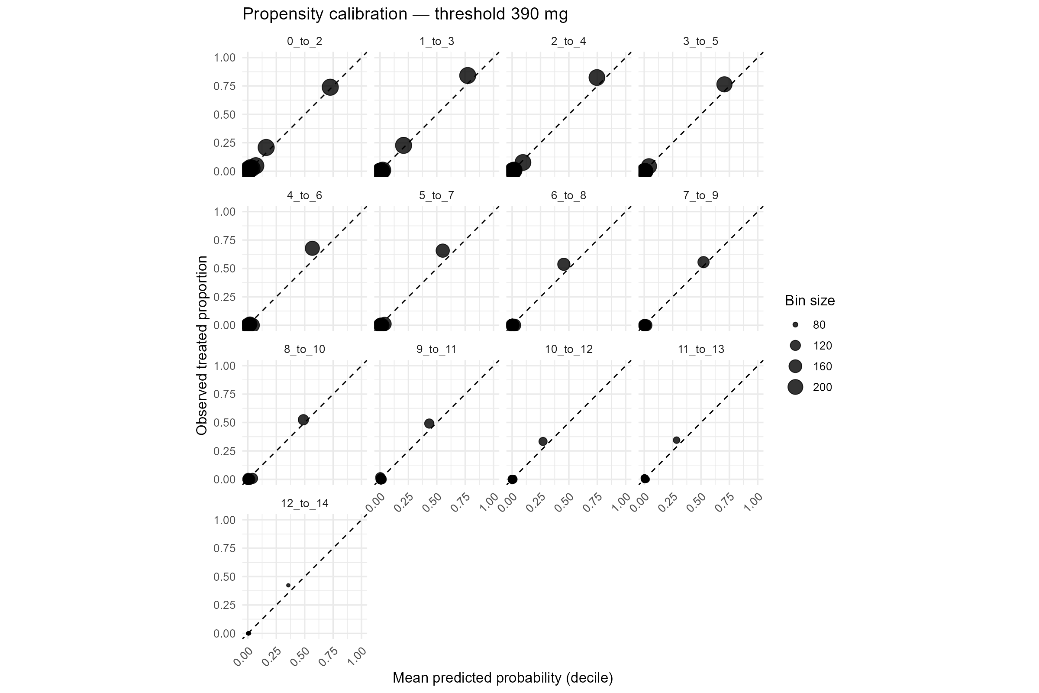

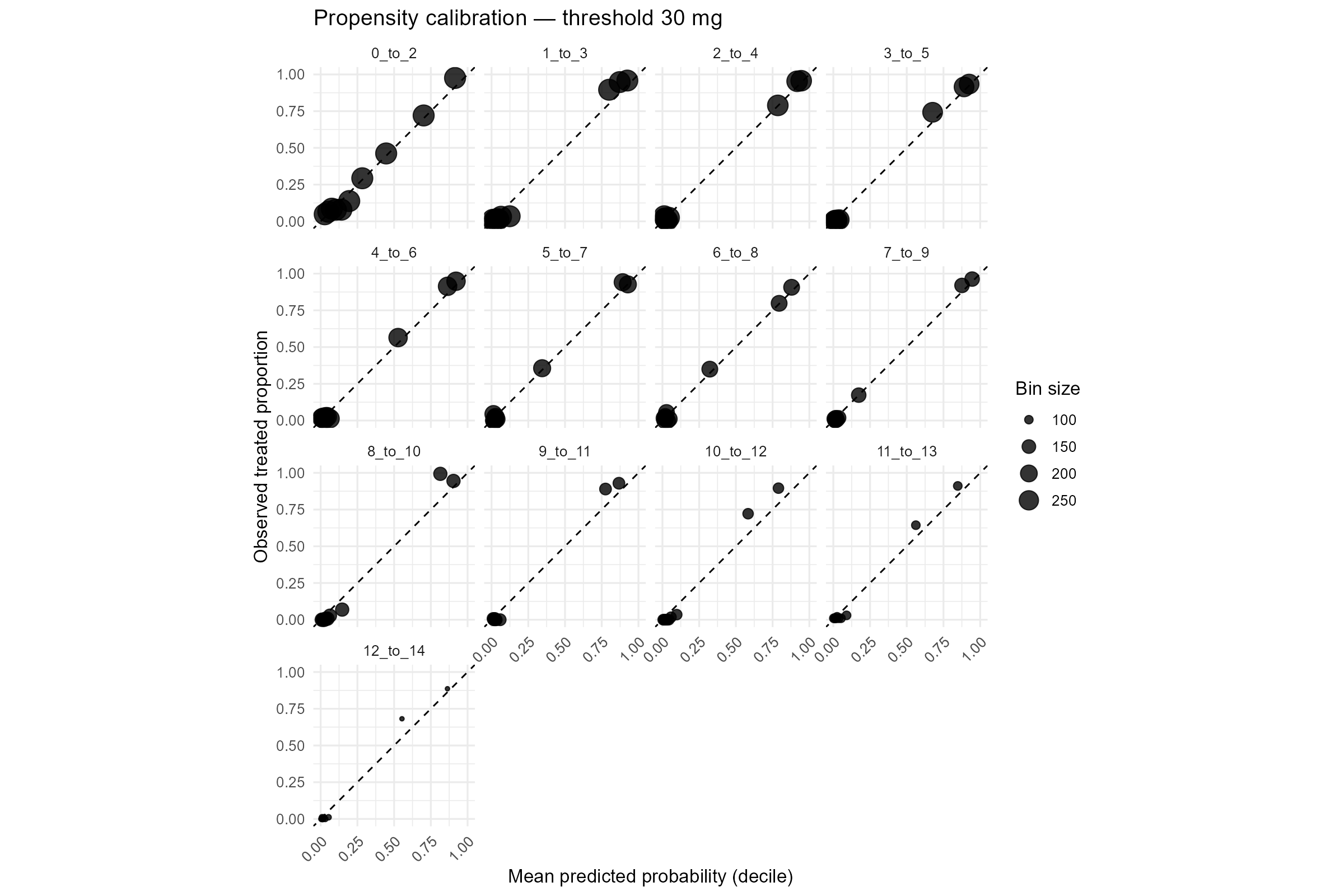

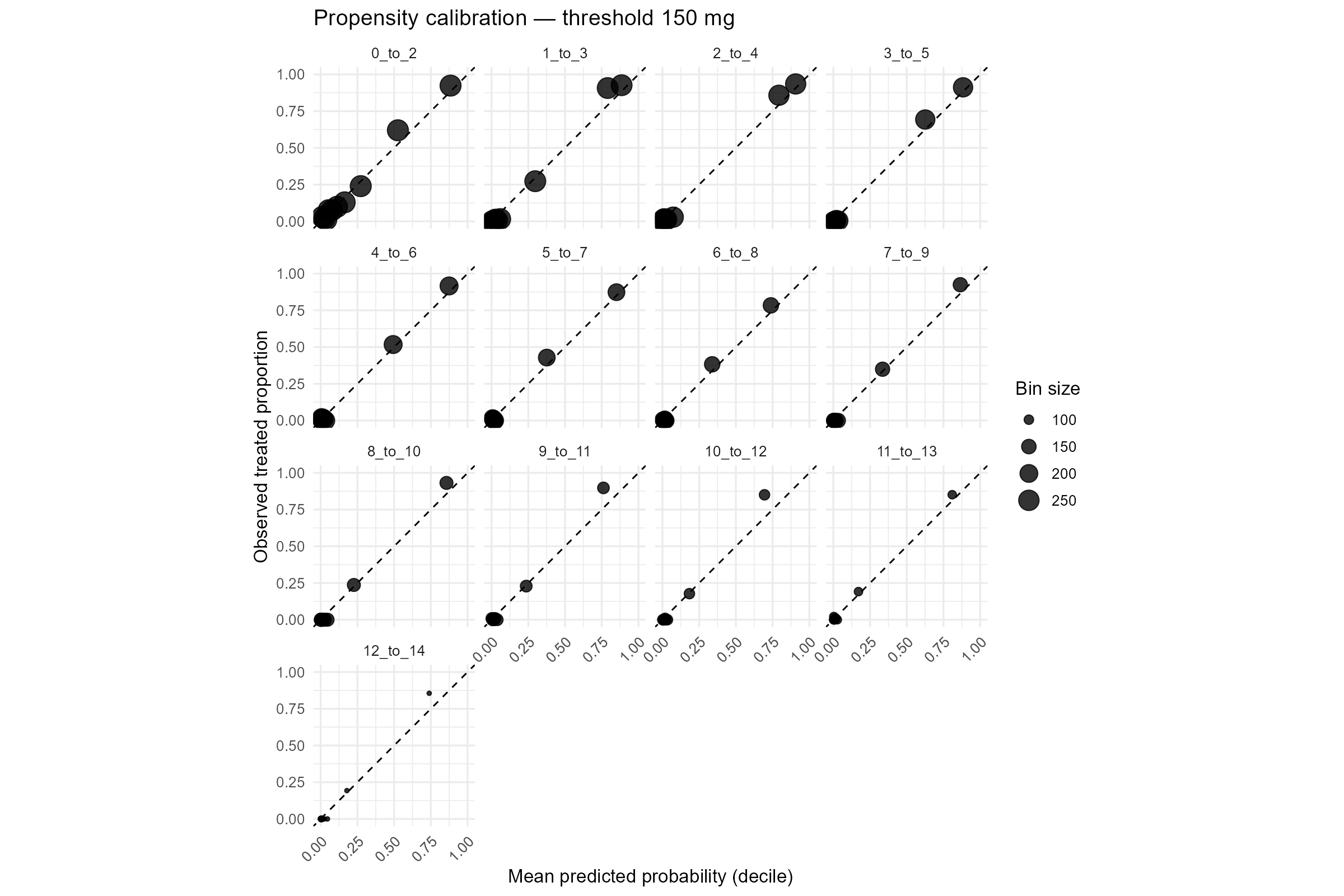

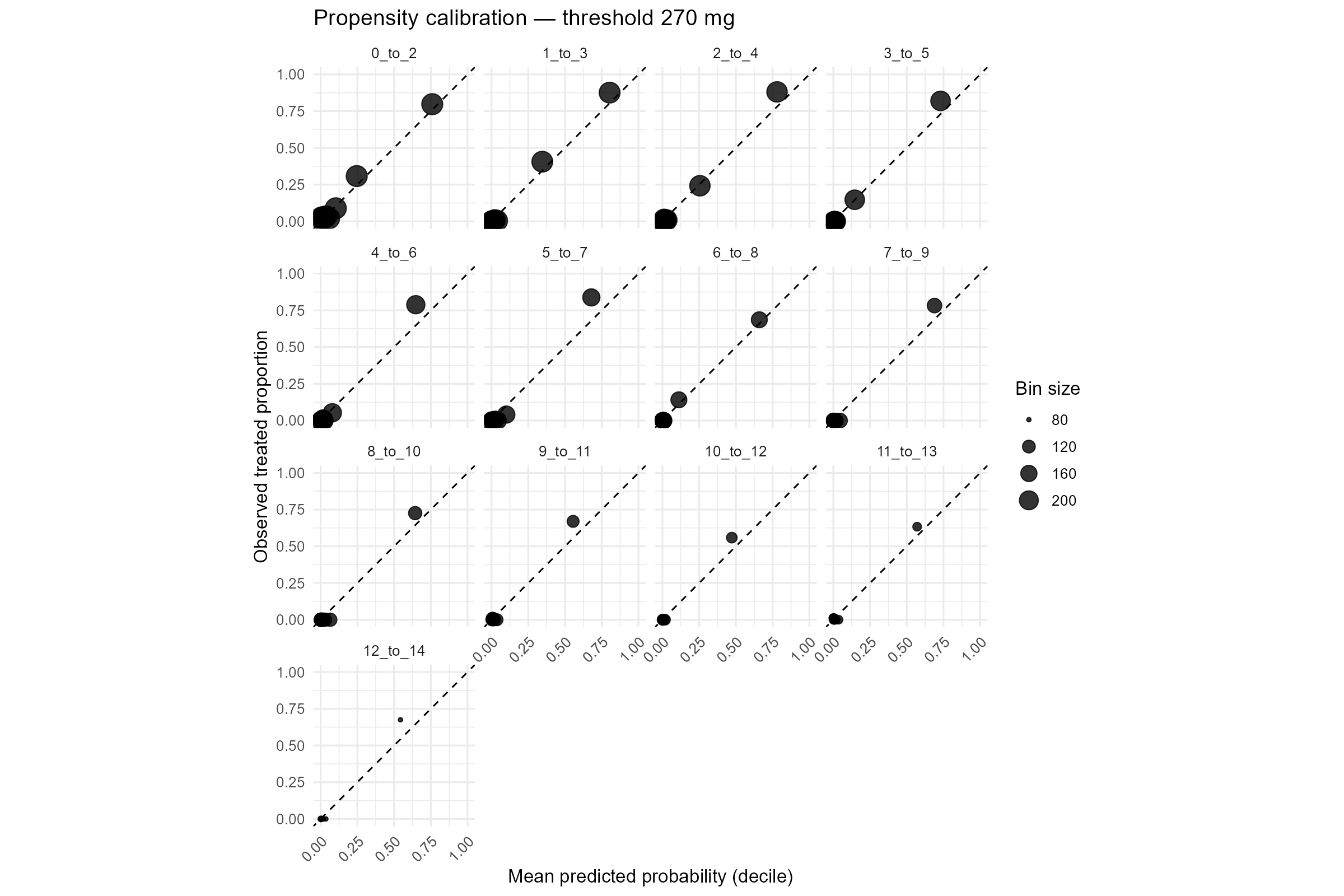


**Figure S5.** Individualised Predicted Risk Difference (PRD) densities by time window.
Kernel density plots of the estimated PRD (risk difference in hospital mortality for treated minus untreated) are shown for each sliding time window (0–2, 1–3, …, 12–14 days). Curves are overlaid within each panel by corticosteroid dose threshold (≥30, ≥150, ≥270, ≥390 mg prednisolone-equivalent over 3 days), with colors indicating dose. The vertical dashed line marks an PRD of 0 (no difference); positive risk differences indicate higher mortality risk associated with corticosteroids. PRD are derived from the overlap-weighted AIPW outcome model used in the primary analysis; when multiple imputations were available, per-subject PRD were averaged within window/threshold before plotting.


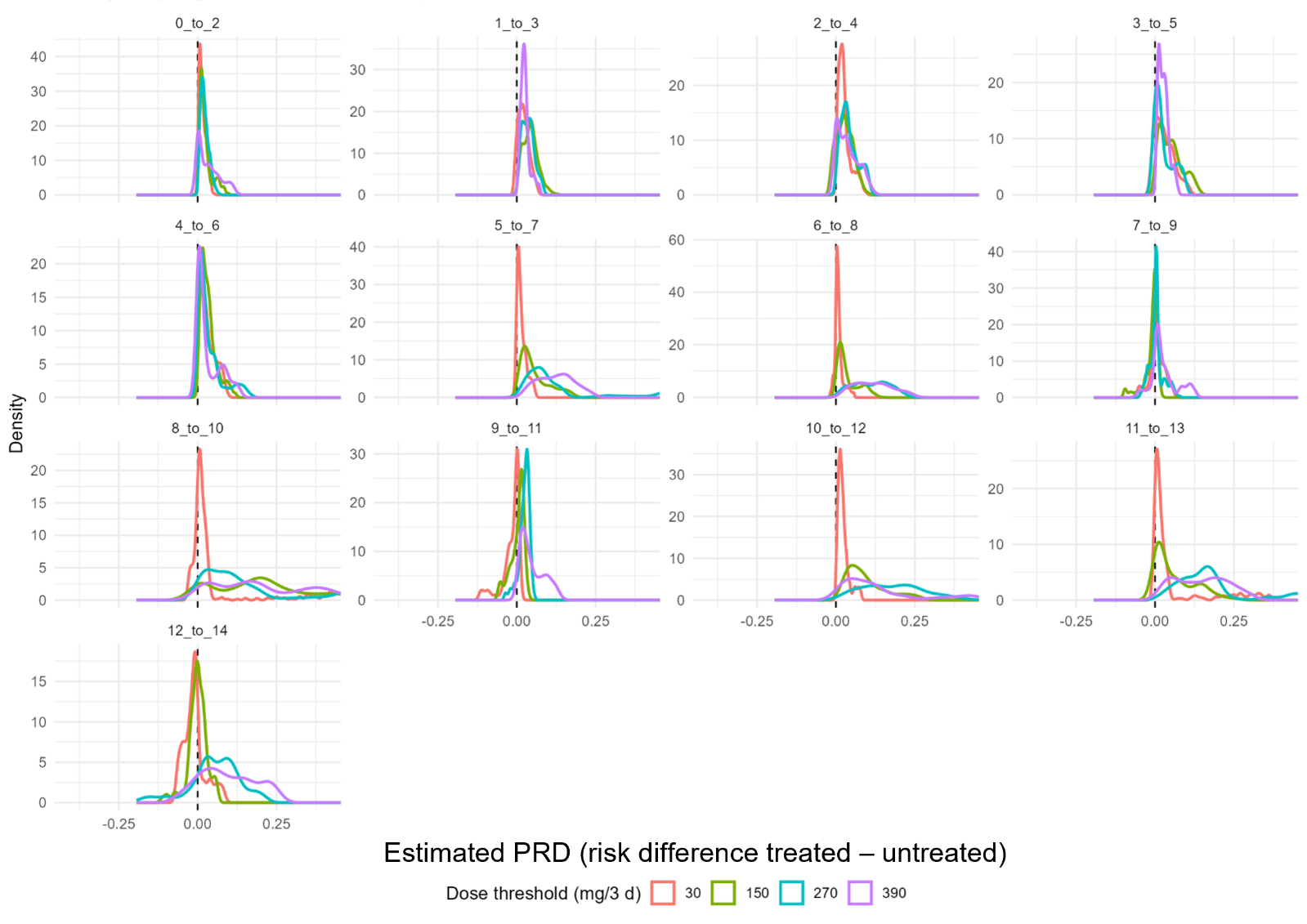


**Figure S6.** Sensitivity to overlap: truncated RD vs OWATE over time
Risk-difference estimates (treated - untreated) across windows comparing the primary overlap-weighted estimand (OWRD) with standard AIPW RD using propensity-score truncation at 0.01 and 0.05. Divergence between curves highlights sensitivity to limited overlap/extreme propensities. Curves are shown for each PE threshold (30, 150, 270, 390 mg/3d); primary cohort and pooled imputations.


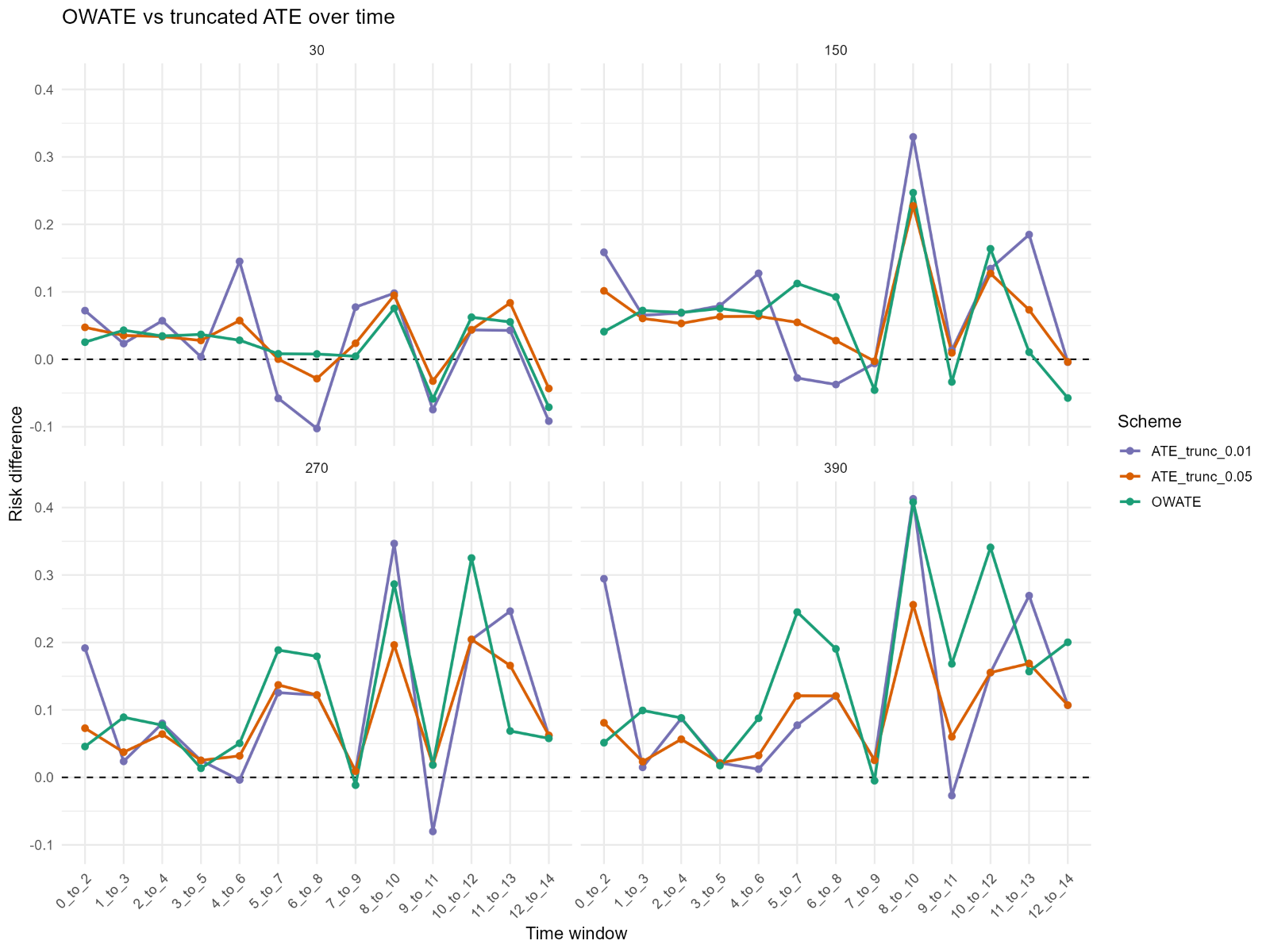


**Figure S7:** Sensitivity to exclusions of pulse-dose steroids and shock. Four-panel layout of overlap-weighted AIPW risk-difference (treated − untreated) estimates for hospital mortality across overlapping 3-day windows, with line/point series colored by corticosteroid dose threshold (prednisolone-equivalent ≥30, ≥150, ≥270, ≥390 mg per 3 days). Panels: top-left primary analysis (no exclusions); top-right pulse excluded; bottom-left shock excluded; bottom-right both excluded. *Pulse* defined as any 24-hour corticosteroid dose >1,000 mg prednisolone-equivalent. *Shock* defined as norepinephrine-equivalent dose ≥0.15 μg/kg/min. Dashed horizontal line marks risk difference = 0. Windows are indexed relative to ARDS onset (0–2, 1–3, …, 12–14 days). Nuisance models are cross-fitted Super Learners, consistent with the primary analysis.


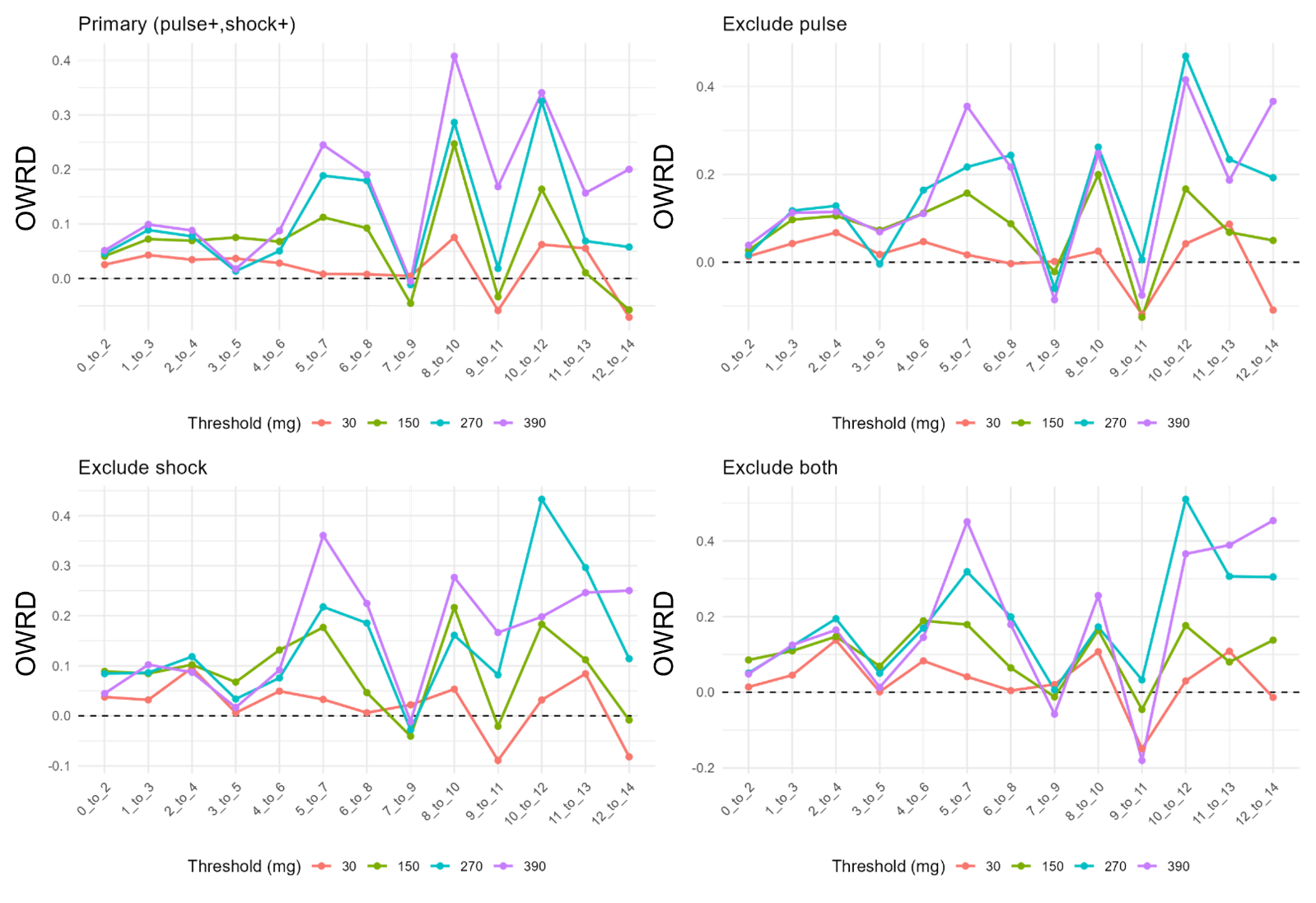


**Table S1**. Covariates used in the propensity-score and outcome models.
Each row lists a candidate covariate; variables appearing in both columns were included in both models, while those appearing in a single column were model-specific. Time-varying summaries are computed within each 3-day window (e.g., 0–2 d, 1–3 d, …) as follows: mean = average across the three days; maximum = highest observed value in the window; range = (maximum − minimum) over the three days; difference = value on the first day minus the value on the last day in the window; variance = within-window variance across the three days. “Corticosteroids in previous window” indicates steroid administration in the immediately preceding 3-day window; “Corticosteroid pre-ARDS onset” indicates exposure before ARDS onset. Diagnosis/comorbidity items (e.g., cancer, pulmonary disease, rheumatic disease, pancreatitis, recent surgery/trauma) are binary indicators derived from baseline data. Abbreviations: P/F, PaO₂/FiO₂ ratio; PaCO₂, arterial carbon dioxide tension; MAP, mean arterial pressure.

| **Propensity Model Covariates** | **Outcome Model Covariates** |
| --- | --- |
| Glucose (mean) | Glucose (mean) |
| Lactate (mean) | Lactate (mean) |
| P/F (mean) | P/F (mean) |
| Peak Inspiratory Pressure (mean) | Peak Inspiratory Pressure (mean) |
| pH (mean) | pH (mean) |
| Charlson Comorbidity Score | Charlson Comorbidity Score |
| Urea (mean) | Urea (mean) |
| Platelet count (mean) | Platelet count (mean) |
| Diagnosis of any Cancer | Diagnosis of any Cancer |
| Diagnosis of Metastatic Cancer | Diagnosis of Metastatic Cancer |
| Temperature (maximum) | Temperature (maximum) |
| Corticosteroids in previous window | Corticosteroids in previous window |
| Corticosteroid pre-ARDS onset | Corticosteroid pre-ARDS onset |
| Surgery | Recent surgery |
| Trauma | Recent trauma |
| Heart rate (range) | Age |
| Diagnosis of Pulmonary disease | Driving pressure (mean) |
| Basophil count (mean) | Lactate (range) |
| Chloride (mean) | Lactate (variance) |
| Eosinophils (mean) | Noradrenaline equivalent (mean) |
| Lactate Dehydrogenase (mean) | Noradrenaline equivalent (range) |
| Potassium (mean) | Peak Inspiratory Pressure (difference) |
| PaCO2 (maximum) | Respiratory rate (mean) |
| Pancreatitis | mean arterial pressure (mean) |
| Diagnosis of Rheumatic disease | Creatinine (mean) |

**Table S2:** Window-level overlap-weighted diagnostics and hospital-mortality estimates, by corticosteroid dose threshold.
Window (overlapping 3-day windows), N (T/C) = total sample with treated/control counts, ESS = stabilized effective sample size under overlap weights (also shown as % of N), Median SMD = median absolute standardized mean difference after weighting, %SMD <0.10 and %SMD<0.20 = proportion of covariates with absolute SMD below 0.10 and 0.20 after weighting, OWRD [95% CI] = overlap-weighted risk-difference estimate for hospital mortality (treated − untreated) with 95% CI, AUC and Brier = out-of-fold discrimination and calibration of the propensity model within each window. Rows are grouped by prednisolone-equivalent dose thresholds (≥30, ≥150, ≥270, ≥390 mg/3 d). Positive RD indicates higher risk associated with corticosteroids. ESS is reported to one decimal and as a percentage of N; RD and CIs are shown as displayed in the table. Abbreviations: OWRD, overlap-weighted risk difference; SMD, standardized mean difference; AUC, area under the ROC curve.

| **Window** | **N (T/C)** | **ESS** | **Median \|SMD\|** | **%\|SMD\|<0.10** | **%\|SMD\|<0.20** | **RD (OWATE) [95% CI]** | **AUC** | **Brier** |
| --- | --- | --- | --- | --- | --- | --- | --- | --- |
| **30 mg/3d** | | | | | | | | |
| 0_to_2 | 968 (285/683) | 480.7 (50%) | 0.047 | 85% | 94% | 0.025 [-0.048, 0.098] | 0.877 | 0.113 |
| 1_to_3 | 961 (280/681) | 272.7 (28%) | 0.113 | 38% | 77% | 0.043 [-0.042, 0.128] | 0.978 | 0.031 |
| 2_to_4 | 935 (264/671) | 154.6 (17%) | 0.133 | 36% | 82% | 0.035 [-0.088, 0.157] | 0.964 | 0.03 |
| 3_to_5 | 866 (228/638) | 159.3 (18%) | 0.125 | 41% | 83% | 0.037 [-0.081, 0.155] | 0.979 | 0.024 |
| 4_to_6 | 774 (197/577) | 177.0 (23%) | 0.091 | 55% | 80% | 0.028 [-0.082, 0.139] | 0.965 | 0.035 |
| 5_to_7 | 685 (159/526) | 119.1 (17%) | 0.19 | 32% | 53% | 0.008 [-0.095, 0.112] | 0.962 | 0.025 |
| 6_to_8 | 610 (133/477) | 140.5 (23%) | 0.088 | 56% | 74% | 0.008 [-0.096, 0.111] | 0.95 | 0.044 |
| 7_to_9 | 540 (115/425) | 82.5 (15%) | 0.203 | 30% | 47% | 0.005 [-0.174, 0.183] | 0.976 | 0.022 |
| 8_to_10 | 485 (99/386) | 147.8 (30%) | 0.136 | 35% | 67% | 0.075 [-0.044, 0.195] | 0.989 | 0.016 |
| 9_to_11 | 426 (78/348) | 98.9 (23%) | 0.226 | 23% | 38% | -0.059 [-0.201, 0.084] | 0.983 | 0.018 |
| 10_to_12 | 385 (64/321) | 111.9 (29%) | 0.15 | 29% | 58% | 0.062 [-0.051, 0.176] | 0.985 | 0.031 |
| 11_to_13 | 337 (56/281) | 81.5 (24%) | 0.214 | 17% | 42% | 0.055 [-0.097, 0.208] | 0.964 | 0.031 |
| 12_to_14 | 296 (47/249) | 58.8 (20%) | 0.202 | 24% | 50% | -0.071 [-0.215, 0.073] | 0.98 | 0.02 |
| **150 mg/3d** | | | | | | | | |
| 0_to_2 | 878 (195/683) | 338.7 (39%) | 0.049 | 77% | 100% | 0.041 [-0.052, 0.134] | 0.897 | 0.089 |
| 1_to_3 | 866 (185/681) | 223.6 (26%) | 0.127 | 42% | 74% | 0.072 [-0.015, 0.159] | 0.985 | 0.027 |
| 2_to_4 | 826 (155/671) | 138.7 (17%) | 0.12 | 47% | 79% | 0.069 [-0.063, 0.201] | 0.974 | 0.025 |
| 3_to_5 | 761 (123/638) | 121.3 (16%) | 0.142 | 36% | 70% | 0.075 [-0.066, 0.217] | 0.988 | 0.023 |
| 4_to_6 | 679 (102/577) | 104.8 (15%) | 0.123 | 32% | 76% | 0.068 [-0.088, 0.223] | 0.961 | 0.03 |
| 5_to_7 | 608 (82/526) | 91.1 (15%) | 0.229 | 18% | 39% | 0.112 [-0.017, 0.242] | 0.964 | 0.021 |
| 6_to_8 | 541 (64/477) | 98.1 (18%) | 0.14 | 30% | 64% | 0.092 [-0.048, 0.233] | 0.973 | 0.033 |
| 7_to_9 | 487 (62/425) | 75.8 (16%) | 0.188 | 38% | 50% | -0.046 [-0.220, 0.129] | 0.994 | 0.015 |
| 8_to_10 | 437 (51/386) | 71.9 (16%) | 0.257 | 26% | 42% | 0.247 [0.107, 0.387] | 0.993 | 0.014 |
| 9_to_11 | 393 (45/348) | 76.4 (19%) | 0.156 | 41% | 55% | -0.034 [-0.188, 0.121] | 0.98 | 0.02 |
| 10_to_12 | 358 (37/321) | 66.7 (19%) | 0.18 | 15% | 61% | 0.164 [0.006, 0.321] | 0.985 | 0.025 |
| 11_to_13 | 315 (34/281) | 42.5 (14%) | 0.237 | 12% | 27% | 0.011 [-0.227, 0.248] | 0.964 | 0.023 |
| 12_to_14 | 278 (29/249) | 52.5 (19%) | 0.256 | 20% | 39% | -0.057 [-0.204, 0.089] | 0.991 | 0.021 |
| **270 mg/3d** | | | | | | | |  |
| 0_to_2 | 791 (108/683) | 184.9 (23%) | 0.063 | 77% | 95% | 0.046 [-0.088, 0.179] | 0.904 | 0.058 |
| 1_to_3 | 782 (101/681) | 140.7 (18%) | 0.158 | 29% | 64% | 0.089 [-0.038, 0.216] | 0.988 | 0.024 |
| 2_to_4 | 759 (88/671) | 123.3 (16%) | 0.12 | 44% | 67% | 0.077 [-0.053, 0.207] | 0.977 | 0.024 |
| 3_to_5 | 706 (68/638) | 104.3 (15%) | 0.233 | 20% | 39% | 0.013 [-0.135, 0.162] | 0.989 | 0.021 |
| 4_to_6 | 631 (54/577) | 97.3 (15%) | 0.19 | 21% | 52% | 0.050 [-0.096, 0.197] | 0.982 | 0.024 |
| 5_to_7 | 577 (51/526) | 105.7 (18%) | 0.173 | 21% | 56% | 0.189 [0.052, 0.325] | 0.99 | 0.02 |
| 6_to_8 | 520 (43/477) | 67.1 (13%) | 0.162 | 38% | 56% | 0.179 [0.018, 0.341] | 0.982 | 0.028 |
| 7_to_9 | 461 (36/425) | 53.8 (12%) | 0.22 | 18% | 45% | -0.012 [-0.183, 0.160] | 0.995 | 0.014 |
| 8_to_10 | 416 (30/386) | 50.0 (12%) | 0.256 | 17% | 38% | 0.286 [0.080, 0.493] | 0.994 | 0.015 |
| 9_to_11 | 374 (26/348) | 48.8 (13%) | 0.246 | 24% | 38% | 0.018 [-0.227, 0.264] | 0.965 | 0.02 |
| 10_to_12 | 340 (19/321) | 33.3 (10%) | 0.22 | 26% | 50% | 0.325 [0.040, 0.610] | 0.985 | 0.021 |
| 11_to_13 | 301 (20/281) | 30.8 (10%) | 0.222 | 26% | 42% | 0.069 [-0.257, 0.394] | 0.948 | 0.025 |
| 12_to_14 | 267 (18/249) | 32.9 (12%) | 0.291 | 5% | 21% | 0.058 [-0.162, 0.278] | 0.992 | 0.017 |
| **390 mg/3d** | | | | | | | |  |
| 0_to_2 | 768 (85/683) | 138.5 (18%) | 0.083 | 64% | 98% | 0.051 [-0.118, 0.221] | 0.927 | 0.045 |
| 1_to_3 | 763 (82/681) | 115.3 (15%) | 0.195 | 38% | 52% | 0.099 [-0.047, 0.245] | 0.99 | 0.022 |
| 2_to_4 | 740 (69/671) | 96.7 (13%) | 0.15 | 35% | 64% | 0.088 [-0.071, 0.247] | 0.976 | 0.023 |
| 3_to_5 | 694 (56/638) | 78.6 (11%) | 0.195 | 26% | 53% | 0.017 [-0.169, 0.204] | 0.988 | 0.02 |
| 4_to_6 | 620 (43/577) | 82.6 (13%) | 0.204 | 27% | 53% | 0.088 [-0.066, 0.241] | 0.985 | 0.022 |
| 5_to_7 | 564 (38/526) | 68.2 (12%) | 0.244 | 20% | 36% | 0.245 [0.047, 0.443] | 0.988 | 0.018 |
| 6_to_8 | 504 (27/477) | 48.5 (10%) | 0.199 | 26% | 47% | 0.190 [-0.028, 0.409] | 0.986 | 0.022 |
| 7_to_9 | 450 (25/425) | 36.3 (8%) | 0.28 | 23% | 36% | -0.005 [-0.277, 0.267] | 0.995 | 0.013 |
| 8_to_10 | 408 (22/386) | 33.0 (8%) | 0.262 | 14% | 38% | 0.408 [0.124, 0.691] | 0.986 | 0.016 |
| 9_to_11 | 367 (19/348) | 34.3 (9%) | 0.246 | 17% | 39% | 0.168 [-0.148, 0.484] | 0.951 | 0.017 |
| 10_to_12 | 332 (11/321) | 20.9 (6%) | 0.356 | 18% | 33% | 0.341 [-0.082, 0.764] | 0.986 | 0.019 |
| 11_to_13 | 292 (11/281) | 22.5 (8%) | 0.324 | 15% | 35% | 0.157 [-0.209, 0.523] | 0.923 | 0.017 |
| 12_to_14 | 260 (11/249) | 18.6 (7%) | 0.393 | 17% | 35% | 0.200 [-0.151, 0.551] | 0.99 | 0.015 |

**Table S3:** Window-level OWRD estimates under four analytic scenarios, by dose threshold.
Tables is divided by dose - ≥30, ≥150, ≥270, and ≥390 mg prednisolone-equivalent over 3 days. Rows are overlapping 3-day windows (e.g., 0–2, 1–3 … 12–14). Columns report, for each window: n_treated and n_control in the analytic sample; OWRD (Primary) = overlap-weighted risk difference for hospital mortality (treated − untreated) with 95% CI shown); ESS = stabilized effective sample size under overlap weights; and the same pair of columns after (i) Pulse excluded (removing stays with pulse-dose corticosteroid days, defined as >1,000 mg methylprednisolone-equivalent within 24 h), (ii) Shock excluded (removing windows with norepinephrine-equivalent dose ≥0.15 μg/kg/min), and (iii) Both excluded. Positive risk differences indicate higher mortality risk under corticosteroids. All quantities are computed independently within each window and dose threshold.

| **30 mg/3d** | | | | | | | | | | |
| --- | --- | --- | --- | --- | --- | --- | --- | --- | --- | --- |
| Window | n_treated | n_control | OWRD (Primary) | ESS (Primary) | OWATE (Pulse excluded) | ESS (Pulse excluded) | OWRD (Shock excluded) | ESS (Shock excluded) | OWRD (Both excluded) | ESS (Both excluded) |
| 0_to_2 | 285 | 683 | 0.03 (-0.05-0.10) | 480.7 | 0.01 (-0.06-0.09) | 465.2 | 0.04 (-0.06-0.14) | 274.5 | 0.01 (-0.08-0.11) | 261.2 |
| 1_to_3 | 280 | 681 | 0.04 (-0.04-0.13) | 272.7 | 0.04 (-0.05-0.13) | 246.7 | 0.03 (-0.08-0.14) | 164.4 | 0.05 (-0.08-0.17) | 132 |
| 2_to_4 | 264 | 671 | 0.03 (-0.09-0.16) | 154.6 | 0.07 (-0.06-0.19) | 143.7 | 0.10 (-0.05-0.24) | 121.4 | 0.14 (-0.01-0.29) | 116.4 |
| 3_to_5 | 228 | 638 | 0.04 (-0.08-0.15) | 159.3 | 0.02 (-0.12-0.15) | 132 | 0.01 (-0.11-0.12) | 157.9 | 0.00 (-0.11-0.11) | 157.3 |
| 4_to_6 | 197 | 577 | 0.03 (-0.08-0.14) | 177 | 0.05 (-0.08-0.17) | 147.8 | 0.05 (-0.05-0.15) | 176.9 | 0.08 (-0.04-0.21) | 142.1 |
| 5_to_7 | 159 | 526 | 0.01 (-0.10-0.11) | 119.1 | 0.02 (-0.08-0.11) | 107.5 | 0.03 (-0.07-0.13) | 106.7 | 0.04 (-0.05-0.13) | 89.8 |
| 6_to_8 | 133 | 477 | 0.01 (-0.10-0.11) | 140.5 | -0.00 (-0.10-0.10) | 141.6 | 0.01 (-0.10-0.11) | 138.6 | 0.00 (-0.11-0.12) | 121.4 |
| 7_to_9 | 115 | 425 | 0.00 (-0.17-0.18) | 82.5 | 0.00 (-0.18-0.18) | 81.8 | 0.02 (-0.13-0.18) | 91.8 | 0.02 (-0.16-0.20) | 82.7 |
| 8_to_10 | 99 | 386 | 0.08 (-0.04-0.19) | 147.8 | 0.03 (-0.08-0.13) | 152.3 | 0.05 (-0.06-0.17) | 163.2 | 0.11 (-0.03-0.25) | 163.9 |
| 9_to_11 | 78 | 348 | -0.06 (-0.20-0.08) | 98.9 | -0.12 (-0.23--0.00) | 132.4 | -0.09 (-0.25-0.07) | 85.6 | -0.15 (-0.34-0.04) | 78.7 |
| 10_to_12 | 64 | 321 | 0.06 (-0.05-0.18) | 111.9 | 0.04 (-0.08-0.16) | 105.7 | 0.03 (-0.10-0.16) | 116.4 | 0.03 (-0.11-0.17) | 102.2 |
| 11_to_13 | 56 | 281 | 0.06 (-0.10-0.21) | 81.5 | 0.09 (-0.10-0.27) | 67.1 | 0.08 (-0.08-0.25) | 80.3 | 0.11 (-0.10-0.32) | 60.5 |
| 12_to_14 | 47 | 249 | -0.07 (-0.22-0.07) | 58.8 | -0.11 (-0.26-0.04) | 49.7 | -0.08 (-0.24-0.08) | 57 | -0.01 (-0.21-0.18) | 51.1 |
| **150 mg/3d** | | | | | | | | | | |
| 0_to_2 | 195 | 683 | 0.04 (-0.05-0.13) | 338.7 | 0.03 (-0.07-0.13) | 312.7 | 0.09 (-0.02-0.20) | 215.2 | 0.09 (-0.04-0.21) | 195.4 |
| 1_to_3 | 185 | 681 | 0.07 (-0.01-0.16) | 223.6 | 0.10 (0.01-0.18) | 226.5 | 0.08 (-0.01-0.18) | 175.9 | 0.11 (0.01-0.21) | 169.8 |
| 2_to_4 | 155 | 671 | 0.07 (-0.06-0.20) | 138.7 | 0.11 (-0.04-0.25) | 125.3 | 0.10 (-0.06-0.26) | 99.2 | 0.15 (-0.01-0.30) | 101.1 |
| 3_to_5 | 123 | 638 | 0.08 (-0.07-0.22) | 121.3 | 0.07 (-0.08-0.22) | 109.4 | 0.07 (-0.06-0.20) | 124.4 | 0.07 (-0.08-0.22) | 105.3 |
| 4_to_6 | 102 | 577 | 0.07 (-0.09-0.22) | 104.8 | 0.11 (-0.04-0.27) | 95.2 | 0.13 (-0.01-0.27) | 104.2 | 0.19 (0.02-0.36) | 80.7 |
| 5_to_7 | 82 | 526 | 0.11 (-0.02-0.24) | 91.1 | 0.16 (0.03-0.28) | 84.5 | 0.18 (0.04-0.32) | 66.4 | 0.18 (0.06-0.30) | 50.1 |
| 6_to_8 | 64 | 477 | 0.09 (-0.05-0.23) | 98.1 | 0.09 (-0.07-0.24) | 82.5 | 0.05 (-0.10-0.19) | 93.3 | 0.06 (-0.11-0.24) | 77.5 |
| 7_to_9 | 62 | 425 | -0.05 (-0.22-0.13) | 75.8 | -0.02 (-0.18-0.14) | 82.8 | -0.04 (-0.20-0.12) | 77.8 | -0.01 (-0.20-0.18) | 59.9 |
| 8_to_10 | 51 | 386 | 0.25 (0.11-0.39) | 71.9 | 0.20 (0.06-0.34) | 106.1 | 0.22 (0.02-0.41) | 116.2 | 0.16 (0.04-0.29) | 116.4 |
| 9_to_11 | 45 | 348 | -0.03 (-0.19-0.12) | 76.4 | -0.13 (-0.33-0.07) | 63.8 | -0.02 (-0.20-0.16) | 72 | -0.05 (-0.30-0.21) | 48.3 |
| 10_to_12 | 37 | 321 | 0.16 (0.01-0.32) | 66.7 | 0.17 (-0.02-0.35) | 51.2 | 0.18 (0.01-0.35) | 63.6 | 0.18 (-0.02-0.38) | 52.3 |
| 11_to_13 | 34 | 281 | 0.01 (-0.23-0.25) | 42.5 | 0.07 (-0.21-0.35) | 36.1 | 0.11 (-0.09-0.31) | 50.8 | 0.08 (-0.13-0.29) | 47.2 |
| 12_to_14 | 29 | 249 | -0.06 (-0.20-0.09) | 52.5 | 0.05 (-0.10-0.20) | 45.9 | -0.01 (-0.20-0.19) | 39.4 | 0.14 (-0.03-0.30) | 62.7 |
| **270 mg/3d** | | | | | | | | | | |
| 0_to_2 | 108 | 683 | 0.05 (-0.09-0.18) | 184.9 | 0.02 (-0.13-0.17) | 158.9 | 0.08 (-0.07-0.23) | 143.1 | 0.05 (-0.10-0.20) | 131 |
| 1_to_3 | 101 | 681 | 0.09 (-0.04-0.22) | 140.7 | 0.12 (-0.02-0.25) | 117.6 | 0.09 (-0.04-0.21) | 123.2 | 0.12 (-0.03-0.27) | 95.8 |
| 2_to_4 | 88 | 671 | 0.08 (-0.05-0.21) | 123.3 | 0.13 (-0.01-0.27) | 110.7 | 0.12 (-0.04-0.27) | 101.5 | 0.19 (0.02-0.37) | 81.3 |
| 3_to_5 | 68 | 638 | 0.01 (-0.13-0.16) | 104.3 | -0.00 (-0.22-0.21) | 72.4 | 0.03 (-0.14-0.20) | 91 | 0.05 (-0.20-0.30) | 58.7 |
| 4_to_6 | 54 | 577 | 0.05 (-0.10-0.20) | 97.3 | 0.16 (-0.03-0.36) | 61.4 | 0.08 (-0.07-0.22) | 92.6 | 0.17 (-0.06-0.40) | 55.5 |
| 5_to_7 | 51 | 526 | 0.19 (0.05-0.33) | 105.7 | 0.22 (0.06-0.37) | 70.4 | 0.22 (0.06-0.38) | 73.2 | 0.32 (0.14-0.50) | 47.1 |
| 6_to_8 | 43 | 477 | 0.18 (0.02-0.34) | 67.1 | 0.24 (0.07-0.42) | 55.7 | 0.19 (-0.01-0.38) | 60.2 | 0.20 (-0.00-0.40) | 51.2 |
| 7_to_9 | 36 | 425 | -0.01 (-0.18-0.16) | 53.8 | -0.06 (-0.41-0.29) | 39.4 | -0.03 (-0.28-0.23) | 37.6 | 0.01 (-0.29-0.31) | 35 |
| 8_to_10 | 30 | 386 | 0.29 (0.08-0.49) | 50 | 0.26 (0.04-0.48) | 39.5 | 0.16 (-0.00-0.32) | 80.2 | 0.17 (-0.01-0.36) | 51.9 |
| 9_to_11 | 26 | 348 | 0.02 (-0.23-0.26) | 48.8 | 0.01 (-0.32-0.33) | 25.3 | 0.08 (-0.18-0.35) | 44.9 | 0.03 (-0.26-0.33) | 29.5 |
| 10_to_12 | 19 | 321 | 0.33 (0.04-0.61) | 33.3 | 0.47 (0.13-0.81) | 22.8 | 0.43 (0.12-0.75) | 30.3 | 0.51 (0.12-0.90) | 22.6 |
| 11_to_13 | 20 | 281 | 0.07 (-0.26-0.39) | 30.8 | 0.23 (-0.14-0.61) | 24.9 | 0.30 (-0.00-0.59) | 31.4 | 0.31 (-0.03-0.64) | 25.7 |
| 12_to_14 | 18 | 249 | 0.06 (-0.16-0.28) | 32.9 | 0.19 (-0.05-0.44) | 25.8 | 0.11 (-0.17-0.40) | 29.5 | 0.30 (0.08-0.53) | 24 |
| **390 mg/3d** | | | | | | | | | | |
| 0_to_2 | 85 | 683 | 0.05 (-0.12-0.22) | 138.5 | 0.04 (-0.14-0.22) | 115.9 | 0.04 (-0.13-0.21) | 119.1 | 0.05 (-0.14-0.23) | 99 |
| 1_to_3 | 82 | 681 | 0.10 (-0.05-0.25) | 115.3 | 0.11 (-0.05-0.27) | 90 | 0.10 (-0.06-0.27) | 92.2 | 0.12 (-0.08-0.33) | 65.9 |
| 2_to_4 | 69 | 671 | 0.09 (-0.07-0.25) | 96.7 | 0.11 (-0.06-0.29) | 91.3 | 0.09 (-0.10-0.28) | 75.1 | 0.16 (-0.05-0.38) | 64.6 |
| 3_to_5 | 56 | 638 | 0.02 (-0.17-0.20) | 78.6 | 0.07 (-0.17-0.31) | 61.6 | 0.02 (-0.17-0.21) | 78.9 | 0.01 (-0.22-0.25) | 55.2 |
| 4_to_6 | 43 | 577 | 0.09 (-0.07-0.24) | 82.6 | 0.11 (-0.10-0.32) | 53.6 | 0.09 (-0.08-0.26) | 63.6 | 0.14 (-0.11-0.40) | 47 |
| 5_to_7 | 38 | 526 | 0.25 (0.05-0.44) | 68.2 | 0.36 (0.11-0.60) | 47.9 | 0.36 (0.12-0.60) | 53.1 | 0.45 (0.20-0.70) | 34.7 |
| 6_to_8 | 27 | 477 | 0.19 (-0.03-0.41) | 48.5 | 0.22 (-0.03-0.47) | 36.1 | 0.22 (0.02-0.43) | 48.5 | 0.18 (-0.15-0.51) | 30.9 |
| 7_to_9 | 25 | 425 | -0.00 (-0.28-0.27) | 36.3 | -0.09 (-0.54-0.37) | 25.2 | -0.01 (-0.29-0.26) | 33.8 | -0.06 (-0.43-0.31) | 23.5 |
| 8_to_10 | 22 | 386 | 0.41 (0.12-0.69) | 33 | 0.25 (-0.00-0.50) | 27.1 | 0.28 (0.10-0.45) | 56 | 0.26 (-0.05-0.56) | 28.6 |
| 9_to_11 | 19 | 348 | 0.17 (-0.15-0.48) | 34.3 | -0.07 (-0.54-0.39) | 18.9 | 0.17 (-0.21-0.55) | 30.2 | -0.18 (-0.78-0.42) | 14.1 |
| 10_to_12 | 11 | 321 | 0.34 (-0.08-0.76) | 20.9 | 0.42 (-0.07-0.90) | 19.7 | 0.20 (-0.30-0.70) | 20.2 | 0.37 (-0.25-0.98) | 18.1 |
| 11_to_13 | 11 | 281 | 0.16 (-0.21-0.52) | 22.5 | 0.19 (-0.28-0.66) | 18.9 | 0.25 (-0.16-0.65) | 20 | 0.39 (0.01-0.77) | 18.2 |
| 12_to_14 | 11 | 249 | 0.20 (-0.15-0.55) | 18.6 | 0.37 (-0.02-0.76) | 14.5 | 0.25 (-0.27-0.77) | 14.1 | 0.45 (-0.01-0.92) | 14.8 |

**Table S3:** Corticosteroid agent use in the ARDS cohort by ICU day (N=987). Values are n (% of total cohort) and median daily dose in mg (IQR). The 'Overall' row shows patients receiving each agent on any day during days 0–13; the dose is the median cumulative actual dose in mg across all days 0–13. ‡ A small number of patients received dexamethasone sodium phosphate (mean dose 17.9 mg IV, n=4) which may reflect neurosurgical rather than ARDS treatment.

| **ICU Day** | **Methylprednisolone** | | **Hydrocortisone** | | **Prednisone** | | **Dexamethasone ‡** | |
| --- | --- | --- | --- | --- | --- | --- | --- | --- |
|  | **n (% of cohort)** | **Median dose, mg (IQR)** | **n (% of cohort)** | **Median dose, mg (IQR)** | **n (% of cohort)** | **Median dose, mg (IQR)** | **n (% of cohort)** | **Median dose, mg (IQR)** |
| **Overall (any day, days 0–13)** | **189 (19.1%)** | **520** | **142 (14.4%)** | **600** | **134 (13.6%)** | **120** | **47 (4.8%)** | **24** |
| Day 0 | 112 (11.3%) | 160 (60–375) | 94 (9.5%) | 200 (100–200) | 53 (5.4%) | 40 (15–60) | 21 (2.1%) | 12 (6–20) |
| Day 1 | 111 (11.2%) | 160 (80–375) | 100 (10.1%) | 200 (152–300) | 42 (4.3%) | 40 (20–60) | 20 (2.0%) | 12 (6–20) |
| Day 2 | 107 (10.8%) | 120 (60–320) | 92 (9.3%) | 200 (152–300) | 51 (5.2%) | 40 (20–60) | 20 (2.0%) | 10 (8–18) |
| Day 3 | 90 (9.1%) | 120 (60–375) | 70 (7.1%) | 200 (140–200) | 57 (5.8%) | 40 (20–60) | 20 (2.0%) | 12 (6–18) |
| Day 4 | 89 (9.0%) | 80 (50–250) | 58 (5.9%) | 150 (100–200) | 60 (6.1%) | 40 (20–60) | 17 (1.7%) | 12 (6–18) |
| Day 5 | 74 (7.5%) | 72 (40–240) | 42 (4.3%) | 100 (52–200) | 58 (5.9%) | 35 (10–60) | 15 (1.5%) | 12 (4–20) |
| Day 6 | 62 (6.3%) | 100 (50–185) | 30 (3.0%) | 100 (52–200) | 52 (5.3%) | 20 (10–40) | 13 (1.3%) | 10 (6–12) |
| Day 7 | 58 (5.9%) | 80 (40–140) | 27 (2.7%) | 100 (52–152) | 49 (5.0%) | 20 (10–60) | 12 (1.2%) | 12 (8–12) |
| Day 8 | 49 (5.0%) | 80 (40–160) | 23 (2.3%) | 100 (52–200) | 54 (5.5%) | 20 (10–60) | 14 (1.4%) | 12 (8–18) |
| Day 9 | 43 (4.4%) | 80 (40–180) | 20 (2.0%) | 100 (52–300) | 44 (4.5%) | 20 (10–60) | 11 (1.1%) | 12 (8–20) |
| Day 10 | 34 (3.4%) | 80 (40–155) | 19 (1.9%) | 200 (52–300) | 37 (3.7%) | 20 (10–40) | 7 (0.7%) | 12 (4–18) |
| Day 11 | 34 (3.4%) | 80 (50–125) | 14 (1.4%) | 200 (52–300) | 26 (2.6%) | 10 (10–40) | 5 (0.5%) | 8 (4–12) |
| Day 12 | 28 (2.8%) | 88 (60–155) | 12 (1.2%) | 150 (76–200) | 20 (2.0%) | 20 (10–30) | 6 (0.6%) | 8 (4–14) |
| Day 13 | 26 (2.6%) | 80 (50–120) | 11 (1.1%) | 150 (100–200) | 15 (1.5%) | 20 (10–30) | 7 (0.7%) | 8 (4–8) |

**Stay_ids for patients in MIMIC-IV Adjudicated as ARDS**

36478457

39032417

35850702

30797241

32925236

32703334

34930825

38715740

37233855

39767392

33409364

30599145

34317198

34785841

33800886

34896096

37896444

33184709

33853395

37430177

35362593

37891482

32399639

34134184

31413176

32762870

30553919

33928870

31328844

39314962

38236345

36310828

33150173

37147149

32125528

38690919

31638539

30697769

33396345

36220002

35296303

38646098

35654318

35486004

39683282

34078574

35739197

32553397

36364051

31545554

35571155

38160524

31138886

36408968

35774507

39432076

34005267

36562457

32069255

33712385

32358573

37287784

39916463

33976845

34857812

31706988

32751084

34197626

31701999

34468245

39672008

30109454

39648536

31548284

31191879

31688799

31090802

36019097

35356603

34763877

32652537

33912654

34680745

38193553

35092097

35888304

39916786

39300189

38165035

30880683

36124281

38253681

33408756

33755900

35342023

30638860

31035340

37364197

33547419

35624477

34168327

30207021

36662174

31663334

31061325

35906940

38712502

31378564

37085556

31793590

39943969

34494950

35043893

34702226

36555100

31303914

30334582

33884628

35377909

34141902

36927864

39626765

37262184

33379036

39606242

37640287

32266418

33404300

32439417

36214523

30542175

35825770

37059308

33367644

36529675

33997173

34890081

30676077

39527567

36784076

31864439

32016049

39110350

38402909

34022066

30596175

39336169

37768645

34223924

38518447

36788229

36139529

38245630

39553351

37193920

36445974

32844650

30267869

36119393

38669895

38326579

35436722

34620357

33747782

36711716

33311865

32964221

33479276

34402429

37920387

33930584

31641592

36270675

38376013

35951426

32994814

30099685

30112339

38157874

32363848

37481477

39841151

32959578

30562036

32092869

36291444

30726244

36231940

31626848

37806558

38115934

39632389

32714013

32374495

32530604

38813583

31940332

38103258

33216558

39005422

33633382

39930837

37970310

35363538

30510323

33514199

39751365

37455091

34901342

34786353

32624981

33843168

31772872

38121875

36162340

36507862

35225836

39048546

39305204

39019105

38844541

32220527

34917578

38875171

33598133

35090797

30385258

39979040

35776742

32347409

39546309

37640039

30339408

34403759

30615140

31872514

30463812

35947837

32354505

36991816

30798940

34776044

37073333

37386043

39067735

34479166

36469158

37331665

30606871

35526828

38366730

30045061

32614649

39434249

32486736

34372594

36360893

30413209

38518443

30344596

34059376

34353289

37257840

31358856

30398810

38368663

38408263

30611160

34749483

36594772

33369539

37970920

39311300

39132620

30650376

38718204

38886396

35467388

37682773

30446809

38948241

37497207

35414722

32293675

33635997

32359580

33032447

36974224

36736089

30254621

33409949

39369124

32462556

32625433

38628629

33742242

36415879

36340090

35118066

38419228

38862706

37924405

37401376

33072687

33592733

31192810

32017381

32086796

37471848

33839862

38272853

31460673

38256401

30691663

36295684

36371437

35384111

36748093

32698525

39093207

38863565

39164071

32900337

38579311

37138898

39428534

31093046

30916660

30575218

31435950

38253505

36045590

31769796

35955882

36364510

33639884

31883804

34708600

33933739

33607465

36740475

38912610

39065777

39788932

34517839

36035672

37222849

31546987

32002159

31300090

36034668

33816926

36357499

37531469

38316442

30495523

32332328

36313464

33085406

38685284

34127111

39748376

38507044

32856031

30297559

33799121

33241111

35642353

36936233

33292851

38092635

39774176

31890769

30169361

37970436

30244643

31302803

31145718

35049634

33507548

33227570

33956085

31314377

38302202

38711594

36260187

36632389

35174400

31574288

30219064

38813138

36427208

32959017

36004625

36328300

39459312

30297632

32482524

30230188

37870474

32688343

37433369

39828578

34198797

39470047

31393514

37080832

37804115

35850696

38714211

33994208

39279323

37122690

32518142

33794426

35449008

34349625

38319736

38660302

38258268

38115015

37679272

30553761

38781322

31011255

34978160

31822408

35763579

36204298

36978490

37424652

31193730

32333651

32349733

39410395

36141845

31532169

33608157

31111301

39998622

30402566

39076122

36662525

31726457

39775416

34557593

30129184

30257407

35718486

34245461

31275316

36738657

34363258

30962143

32013274

37625314

38353532

36532173

39142568

35759680

30791603

34291164

35011237

32408660

33959042

35794404

34575919

30748451

33102854

34988733

31058224

35278428

30111451

34664245

35062531

35558988

35587828

32787832

32642582

35438780

37270064

31647174

33708180

38360523

37064466

32905110

34310308

32105226

34003859

34477454

34451600

36272287

33336719

34331895

35642150

34294325

33594842

38848710

34280776

30063700

30500045

39726152

30563924

32906693

31651106

39635126

34649276

34250110

38839114

33834452

39708226

36530840

37352025

34453158

35545799

36169399

31962750

31149608

33546403

31608691

35826008

37354138

30413290

30027612

30158404

33226289

37805633

34139610

38367142

37404080

33907246

33063599

39008675

35681480

38145651

36434432

33367985

38156998

30244200

30966963

38166577

38310952

31759239

34450694

38502165

39771268

33029615

38424830

39235862

36719684

34147846

35192961

37650707

30832709

34317173

39574750

36278120

36471889

30949248

32380519

37961196

30717105

36450265

38560057

38404233

32561859

33332987

35864252

31605385

38881410

36225739

32740603

36135694

36555862

38690182

38853975

31444035

36917234

34818547

30117582

32795641

30798531

39945913

35313115

31186320

39642689

34963459

39992167

37985659

38928875

34030023

36470971

33316609

39131197

35586100

34033876

34280555

30227036

30529254

37956867

34350939

34711898

31033547

30378899

35305163

32842637

38097393

35388967

32612189

30591925

30459451

31771936

33205866

38548240

33930962

35072949

33198182

39285843

39689646

35056297

37728826

34499406

32505492

37317518

30045625

34925336

31791282

37521453

38044131

33910341

38290392

34609091

33700270

35355731

38985088

34958629

36460006

39806964

34435551

36872985

38544081

37423544

35645575

36898085

39636839

39361421

31898214

36374005

38000007

34400144

32203313

31495164

36218320

34845295

34125208

31797887

33098612

35016614

31340730

30465888

33905921

38504972

30129989

34148423

36740005

39422872

36121575

30407507

35538669

35225810

38995437

34153778

30868865

31777308

30421531

34804855

30066630

33809090

32137898

35145280

39663204

30458688

30748248

33740916

33734794

30249183

33482269

37451821

31294668

30252483

33621002

39441572

30585683

36828715

37927455

35429155

31603200

38205011

36582479

38798172

30545415

35695493

38500204

37708679

31631455

32386822

39690695

34069364

37959296

30645946

30866972

31681715

36145269

37528363

33243793

38859514

31046887

30729765

37082640

35446806

39602181

38852670

35854963

35662928

38911695

36222295

33612889

35245345

38767530

38905861

36362374

30033048

30056659

36049628

31921426

31621601

38465424

30461396

32737479

39506648

36962764

38099719

36535736

32569128

37794348

36016876

32336965

33661789

39380090

30078723

38706653

33865177

30520948

39336911

33485623

33832011

31550648

33641749

33528947

37026266

31814403

35520548

32874601

38892147

34010095

35771738

39000714

37772708

33196019

31616659

36448639

31978081

37800255

35171466

36985041

31517631

31593624

37480685

38462481

31918372

37658939

31863678

34431011

36077460

31583232

33181285

39863507

31265331

31100931

36572137

30588455

39965946

39781184

37601751

36666568

38619741

30613655

34341380

39522266

38373548

38344915

32311012

37287634

37582802

33316407

34053363

30961397

39272324

34919439

34286221

37603454

30146534

36697359

38545284

38021692

36028102

34900629

36078619

36915007

39138759

33964764

39755898

33464058

31180945

33746945

35346352

38535695

39707309

38573575

33336389

38315860

37220696

31132695

34166660

36174467

34016559

39685365

39150002

32490594

35198144

35317152

30907727

31274661

32026882

32523123

32334239

32054985

32962674

34387455

36502457

30630049

35359444

34139557

36787203

30838616

33972413

30421216

32526703

32798636

36952026

31837792

33170405

34409301

30547971

31021231

37119050

35365595

39100453

35963122

34155256

33321724

38447805

36246235

33479900

39488188

34355317

39078312

31859365

39330400

39532341

31583513

39939179

32458586

31534048

38713500

38927418

31438123

38060507

38656987

32897977

35669025

32416354

30828325

33014032

38358573

37767813

32435143

38680413

33949923

32911554

39897084

32220998

34784026

39822017

38197705

36464175

36577023

35292383

38446756

30083424

30809433

30423942

34544862

35490965

35312153

34689947

30912986

37929227

31891369

31725625

35418172

38037144

38322114

34852120

37734919

39167475

31869180

31679114

37512067

33975208

38990966

37284177

34522604

38072803

30481610

34996080

32025098

36408173

33974423

36636407

31367662

32170791

34038447

36335999

39393356

37644513

33976461

37931991

35941893

35856224

33929726

37274881

30468690

36881400

37211192

30225882

36113149

38284226

39357519

37547861

35503068

38989889

30744533

30979781

36827809

36289512

36000041

38712254

31754815

39132344

33042587

35777876

30265100

32668090

38030591

39340451

30039231

39466010

35164439

37747640

34306381

39793001

35817020

30216662

33763417

33538434

31920220

36040375

35623166

32172367

38573398

35970338

37541993
